# Central Nervous System Barriers Impact Distribution and Expression of iNOS and Arginase-1 in Infiltrating Macrophages During Neuroinflammation

**DOI:** 10.3389/fimmu.2021.666961

**Published:** 2021-04-15

**Authors:** Daniela C. Ivan, Sabrina Walthert, Giuseppe Locatelli

**Affiliations:** Theodor Kocher Institute, University Bern, Bern, Switzerland

**Keywords:** macrophage, blood-brain barrier, cell trafficking, iNOS - inducible nitric oxide synthase, arginase 1 (ARG1), choroid plexus (CP), meninges, EAE (experimental autoimmune encephalomyelitis)

## Abstract

In multiple sclerosis (MS) and other neuroinflammatory diseases, monocyte-derived cells (MoCs) traffic through distinct central nervous system (CNS) barriers and gain access to the organ parenchyma exerting detrimental or beneficial functions. How and where these MoCs acquire their different functional commitments during CNS invasion remains however unclear, thus hindering the design of MS treatments specifically blocking detrimental MoC actions. To clarify this issue, we investigated the distribution of iNOS^+^ pro-inflammatory and arginase-1^+^ anti-inflammatory MoCs at the distinct border regions of the CNS in a mouse model of MS. Interestingly, MoCs within perivascular parenchymal spaces displayed a predominant pro-inflammatory phenotype compared to MoCs accumulating at the leptomeninges and at the intraventricular choroid plexus (ChP). Furthermore, in an *in vitro* model, we could observe the general ability of functionally-polarized MoCs to migrate through the ChP epithelial barrier, together indicating the ChP as a potential CNS entry and polarization site for MoCs. Thus, pro- and anti-inflammatory MoCs differentially accumulate at distinct CNS barriers before reaching the parenchyma, but the mechanism for their phenotype acquisition remains undefined. Shedding light on this process, we observed that endothelial (BBB) and epithelial (ChP) CNS barrier cells can directly regulate transcription of *Nos2* (coding for iNOS) and *Arg1* (coding for arginase-1) in interacting MoCs. More specifically, while TNF-α+IFN-γ stimulated BBB cells induced *Nos2* expression in MoCs, IL-1β driven activation of endothelial BBB cells led to a significant upregulation of *Arg1* in MoCs. Supporting this latter finding, less pro-inflammatory MoCs could be found nearby IL1R1^+^ vessels in the mouse spinal cord upon neuroinflammation. Taken together, our data indicate differential distribution of pro- and anti-inflammatory MoCs at CNS borders and highlight how the interaction of MoCs with CNS barriers can significantly affect the functional activation of these CNS-invading MoCs during autoimmune inflammation.

## Introduction

Central nervous system (CNS) barriers comprise cellular and molecular specializations which limit the traffic of pathogens and of blood-borne immune cells toward the organ parenchyma (1). Tight barriers are present at the level of the leptomeningeal vasculature within the subarachnoid space, of the blood-cerebrospinal fluid (CSF) barrier around the choroid plexus (ChP), and of the blood-brain barrier (BBB) in CNS microvascular endothelial cells (2). Passage through these CNS gateways leads to cells accumulating from the BBB in the perivascular space, from the blood-CSF barrier and the leptomeningeal vessels in the cerebrospinal fluid (CSF) and the subarachnoid space (2). These anatomical compartments are separated from the CNS parenchyma by a second “checkpoint” mechanism constituted by the glia limitans (3, 4).

During neuroinflammatory conditions such as multiple sclerosis (MS), different immune cells including monocyte-derived cells (MoCs) cross these barriers and reach the CNS parenchyma, where they guide disease progression (5). This multifocal passage of MoCs through CNS barriers is also a pathological hallmark of experimental autoimmune encephalomyelitis (EAE), a widely-used animal model of MS-like CNS autoimmunity (6).

In both MS and EAE, MoCs are the most abundant immune cells found within inflammatory lesions (7, 8). In the mouse, MoCs arise from circulating Ly6C^high^CX3CR1^low^ monocytes and show fast CCR2-mediated recruitment to inflamed tissues (9, 10). Following CNS infiltration, MoCs exert a wide array of functions ranging from damaging pro-inflammatory to tissue repairing anti-inflammatory actions (11–13).

The remarkable plasticity of MoCs originates from the dynamic integration of numerous local signals (14), with pro- and anti-inflammatory cells characterized by distinct metabolic states and by differential regulation of phenotypic markers (11). Among these, expression of the enzymes inducible nitric oxide synthase (iNOS) and arginase-1 is commonly used as signature marker for MoC pro- and anti-inflammatory polarization, respectively (15). These two proteins utilize L-arginine as substrate for the production of cytotoxic nitric oxide and citrulline (iNOS), or urea and ornithine (arginase-1) with key tissue repair properties (16).

By visualizing the expression of distinct reporter proteins under the control of *Nos2* and *Arg1* promoters in *iNOS-tdTomato x Arginase-EYFP* mice, we have recently described the functional evolution of CNS-invading MoCs in the EAE model (17). During disease development, CCR2^+^iNOS^+^ MoCs (M^iNOS^) formed inflammatory CNS lesions, progressively increased arginase-1 expression (M^iNOS/Arginase^ intermediates) and finally switched their phenotype toward an iNOS^negative^-arginase-1^+^ state (M^Arginase^). This transition was paralleled by the distinct CNS infiltration of M^Arginase^ cells that did not previously express iNOS (17). Notably, even before reaching the CNS parenchyma, MoCs accumulating at CNS barriers could display a complete functional specification which likely contributes to the disease evolution (17).

However, the polarizing factors and the sub-anatomical CNS compartments where MoCs acquire their phenotype are not known. Furthermore, whether pro- and anti-inflammatory MoCs are preferentially recruited at specific CNS gateways remains unclear. This missing information hinders the design of therapeutic interventions potentially blocking the invasion and activation of cytotoxic MoCs during neuroinflammation.

To tackle this problem, we here assessed the anatomical routes of MoCs migration to the CNS and investigated whether their interaction with distinct CNS barriers regulates the functional polarization of invading MoCs.

Notably, in the EAE model we could observe the presence of iNOS^+^ and arginase-1^+^ MoCs within all CNS border areas, with barrier-specific differences showing for instance higher presence of pro-inflammatory M^iNOS^ cells compared to transitional M^iNOS/Arginase^ cells at perivascular spaces of the BBB. In parallel, an *in vitro* model revealed that functionally-polarized MoCs are in principle able to migrate through the blood-CSF barrier of the ChP, together suggesting that the ChP could constitute a CNS access gateway and a polarization site for both pro- and anti-inflammatory MoCs.

Secondly, we assessed whether the interaction with CNS barriers would directly influence the functional state of trafficking MoCs. Interestingly, we observed that IFN-γ-stimulated barrier cells induced a significant upregulation of *Nos2* in interacting MoCs, while *Arg1* induction appeared dependent on IL-1β stimulation of BBB cells. IL-1β signaling at the BBB influenced the phenotype of invading MoC also *in vivo*, as shown by analysis of MoCs around IL1R1^+^ BBB endothelial cells in animals suffering from EAE.

Taken together, our data indicate how the local access gateways and microenvironments utilized by MoCs to access the CNS parenchyma substantially affect their migration and functional specification during autoimmune CNS inflammation.

## Material and Methods

### Animals

C57BL/6J mice were purchased from Janvier (Genest Saint Isle, France). *Arginase EYFP* mice were originally purchased from The Jackson Laboratory, *iNOS-tdTomato* were kindly provided by Prof. Alain Bessis (ENS Paris, France); *CCR2-RFP x CX3CR1-GFP* mice were a gentle gift of Dr. Israel F. Charo (UCSF, USA). *VE-cadherin-GFP* mice were produced and donated by Prof. Dietmar Vestweber (Max Planck Institute Münster, Germany). Mice were housed in individually ventilated cages under specific pathogen-free conditions. Animal procedures were performed in accordance with the Swiss legislation on the protection of animals and were approved by the veterinary office of the Canton of Bern, Switzerland.

### Active Experimental Autoimmune Encephalomyelitis (aEAE) Induction

aEAE was induced by injection of myelin oligodendrocyte glycoprotein peptide 35-55 (MOG_35-55_ peptide, 200 µg per animal, Genscript, USA) and complete Freund’s adjuvant (prepared from Incomplete Freund’s Adjuvant, Santa Cruz Biotechnology, USA; supplemented with Mycobacterium Tubercolosis, Difco). Briefly, an emulsion of MOG_35-55_ and CFA was injected subcutaneously in mouse flanks and at the tail base at day 0; in addition, 300 ng of pertussis toxin (List Biological Laboratories, Campbell, CA, USA) was injected intraperitoneally at day 0 and day 2. Immunized mice were weighted and disease development scored daily according to a previously established system (18). Four time points were defined for analysis: weight loss (animals presenting a 3-5% loss of weight shortly preceding clinical symptoms), day of clinical onset (animals showing a limp tail and partial weakening of hind limbs), symptomatic peak of disease (animals presenting strong hind limb paraparesis or full paraplegia, 3-4 days after EAE onset), and remission (animals showing slight hind limb paraparesis after having displayed hind leg paraplegia, 7-8 days after disease onset).

### Brain Isolation and Vibratome Sections

Mice were sacrificed and transcardially perfused with 2% paraformaldehyde (PFA, Merk Darmstadt, Germany) in Dulbecco´s phosphate- buffered saline (DPBS, Gibco, Paisley, UK); isolated brains were post-fixed in 2% PFA overnight and were embedded in 2% low-melt agarose (Sigma-Aldrich, St. Louis, MO, USA) in DPBS. Brains were sliced coronally at a thickness of 100µm, using a vibratome (VT1000S, Leica Biosystems, Muttenz, Switzerland) at a speed of 0.65 mm/s and a frequency of 80Hz. The samples were collected in ice cold DPBS.

### Spinal Cord Isolation and Cryostat Sections

Mice were sacrificed and transcardially perfused with 4% paraformaldehyde (PFA, Merk Darmstadt, Germany) in Dulbecco´s phosphate- buffered saline (DPBS, Gibco, Paisley, UK); isolated spinal cords were post-fixed in 4% PFA overnight, left for 3 days in 30% sucrose (Sigma-Aldrich, St. Louis, MO, USA) diluted in DPBS and then frozen at -80°C in O.C.T. (Tissue-Tek). 20 to 40 µm spinal cord longitudinal sections were cut using a cryostat (HM550, Thermo Fisher).

### Immunofluorescence Stainings of CNS Tissue

For staining of ChP sections, vibratome-cut free-floating brain slices were initially washed with 1x Tris-Buffered saline (TBS), containing 10x TBS - 50mM Trizma Base (Sigma-Aldrich, St. Louis, MO, USA), 150mM NaCl (Sigma-Aldrich, Buchs, Switzerland) and 1mM CaCl_2_ x 2H_2_O (Sigma-Aldrich, St. Louis, MO, USA), pH 7.4. Slices were incubated with blocking buffer containing TBS with 5% skimmed milk (Rapilait, Migros, Switzerland), 0.3% Triton X-100 (Sigma-Aldrich, St. Louis, MO, USA) and 0.04% NaN_3_ (Fluka Chemie, Buchs, Switzerland), pH 7.4, for 2h at room temperature (RT). To stain the blood vessels, we made use of the MEC13.3 antibody (anti-PECAM-1/CD31, rat IgG2a, home-made), prepared in blocking buffer and incubated overnight at 4°C on a rocker. After rinsing 3 x 5 min with 1x TBS, a secondary Cy5-conjugated AffiniPure donkey anti-rat IgG (H+L) (1:200, stock of 0.5 mg/ml, Jackson ImmunoResearch Laboratories, West Grove, PA, USA, catalog number 712-175-153) was diluted in blocking buffer and applied to the sections for 2h at RT. The slices were incubated with DAPI (1:5000, 1mg/ml stock, AppliChem, Darmstadt, Germany), diluted in 1x TBS for 20 min at RT. After washing and drying, the slices were mounted with Mowiol 4-88 (Aldrich, St Louis, MO, USA).

For staining of cryostat-cut spinal cord sections, slices were initially fixed with 100% ice cold acetone at −20°C for 10min and dried before being reconstituted with 1x Tris-Buffered saline (TBS), containing 10x TBS (see above). We blocked unspecific antibody binding with 10% goat or donkey serum containing 0.1% Triton (Sigma-Aldrich, St. Louis, MO, USA) diluted in TBS for 1h at RT. For primary antibody stainings, we used rabbit anti-laminin (Dako, stock 3.8mg/ml, 1:1000), and goat anti IL1R1 (polyclonal IgG, R&D Systems, stock 0.2mg/ml, 1:100) antibodies prepared in 2% goat or donkey serum, respectively, containing 0.1% Triton in TBS and incubated overnight at 4°C. After rinsing 3 x 5 min with 1x TBS, a secondary goat anti-rabbit AF647 (Invitrogen, stock 2mg/ml, 1:500) or donkey anti-goat AF647 IgG (H+L) (Jackson ImmunoResearch, 1:200) antibody were applied in 2% goat or donkey serum for 2h at RT. Slices were incubated with DAPI (1:5000 in TBS, 1mg/ml stock, AppliChem, Darmstadt, Germany) for 10 min at RT. Slices were mounted with Mowiol 4-88 (Sigma-Aldrich, St Louis, MO, USA).

### Density Analysis of MoCs at CNS Barriers

Z-stack images of CNS sections were acquired using a LSM800 confocal microscope (Zeiss) with 40x magnification, and analyzed using Fiji (National Institute of Health, Bethesda, MD, USA). Sections from C57BL/6J mice (both healthy and at different time-points following EAE induction) were used to infer tissue background and exclude artifacts. In sections from *iNOS-tdTomato x Arginase-EYFP* and *VE-cadherin x iNOS-Tomato x Arginase-EYFP* mice, expression of tdTomato and EYFP was assessed manually and blindly. In sections from *CX3CR1-GFP x CCR2-RFP* mice, numbers of RFP and GFP expressing cells was assessed with a Fiji macro (**Supplementary Data 1**). Cell density in CNS sections was calculated manually on selected z-planes in image stacks using Fiji and extrapolated to cells per mm^2^.

### Bone Marrow-Derived Macrophage Isolation and Cell Culture

The isolation, culture and stimulation of MoCs from the bone marrow were performed according to previously established protocols (17). Briefly, pelvis, tibia and femurs were isolated from seven to twelve weeks-old C57BL/6J male mice. The bone marrow was flushed using RPMI with glutamine (Gibco, Paisley, UK) supplemented with 10% heat inactivated fetal bovine serum gold (FBS, Gibco, Paisley, UK) and 100IU/ml penicillin-streptomycin (Gibco, Paisley, UK) (henceforth called MoC media). Following filtration through 100μm filters (Corning), cells were incubated in 1ml of Ack lysing buffer (Gibco, Grand Island, NY, USA) for 5 min on ice to deplete erythrocytes. After washing, cells were cultured in MoC media supplemented with 5ng/ml recombinant mouse macrophage colony stimulating factor (mCSF, 416-ML-500, R&D Biosystems, Minneapolis, USA) for seven days at 37°C, 5% CO_2_, at a confluence of 2 million cells/ml, in non-treated tissue culture 100mm Petri dishes (Greiner Bio-One, St. Gallen, Switzerland). For *in vitro* migration experiments, at culture day seven, MoCs were polarized for 48h towards: a pro-inflammatory profile (M^LPS+IFN-γ^), with 100ng/ml lipopolysaccharide from *salmonella enterica serotype typhimurium* (LPS, Sigma-Aldrich, St. Louis, MO, USA, catalog number L4516) and 10ng/ml recombinant murine IFN-γ (315-05, PeproTech, Rocky Hill, NJ, USA); an anti-inflammatory phenotype (M^IL-4+IL-13^), with 10ng/ml recombinant murine IL-4 (404-ML, R&D Biosystems, Minneapolis, USA) and 10ng/ml IL-13 (413-ML-025, R&D Biosystems, Minneapolis, USA); or were left unstimulated (M^unpolarized^) in MoC medium containing mCSF. MoCs were collected following 10min incubation in 0.05% Trypsin (25300-054, Gibco, Paisley, UK) at 37°C.

### mRNA Isolation

To isolate mRNA from MoCs, cells were washed with ice cold sterile 1x DPBS and incubated for 5 min in 1ml TRIzol (Invitrogen, Leicestershire, UK) or RNA-Bee (Amsbio, CS-501B, UK) at RT. After cell scraping, a volume of 250μl chloroform (Merck, Darmstadt, Germany) was added. The cells were vigorously shaken for 15 seconds, left at RT for 5min, and centrifuged at a speed of 9300 x g for 15 min at 4°C. The top layer aqueous phase (containing RNA) was carefully extracted and mixed gently with 500μl isopropanol (Grogg Chemie, Stettlen, Switzerland). After 5min incubation and 20min 13400 x g centrifugation at 4°C, the pellet was washed once with ice cold 75% ethanol (Merck, Darmstadt, Germany). After complete ethanol evaporation, the pellet was dissolved in 25μl ultrapure water and the mRNA concentration was measured using a NanoDrop (Thermo Fisher Scientific, Rochester, NY, USA). mRNA purity was assessed using the 260/280 nm and 260/230 nm ratios.

### cDNA Synthesis and RTqPCR

To synthesize the complementary DNA (cDNA) from single stranded mRNA *via* reverse transcription, we used SuperScript™ III Reverse Transcriptase cDNA synthesis kit (Invitrogen, Carlsbad, CA, USA, 18080-051) according to manufacturer´s instructions. Briefly, equal amounts of mRNA from each sample were incubated with 50ng random hexamer primers and 1mM dNTP Mix for 5min at 65°C. The reaction was then allowed to cool on ice for 5min to allow primer binding. Afterwards, a cDNA synthesis mix containing 10x first-strand buffer, 0.1M DTT, 40IU/μl RNAseOUT (Invitrogen, Carlsbad, CA, USA, catalog number 10777-019), and 200IU/μl reverse transcriptase solution was added. The complementary DNA was catalyzed by incubating the samples at 25°C (10min) and 50°C (50min) in a PCR thermal cycler (Mastercycler X59s, Eppendorf, Hauppauge, NY, USA). The reaction was inactivated at 70°C for 15 min and allowed to cool at 4°C for 10min. To perform real time quantitative PCR reaction (RTqPCR), we used Taykon™ Low Rox SYBR MasterMix dTTP blue (Eurogentec, Liege, Belgium) according to manufacturer´s instructions. A total amount of 8.75ng cDNA per well was used (in a total volume of 20ul/well). Each sample was tested in triplicates in MicroAmp™ Optical 384-well reaction plates (Applied Biosystems, Waltham, Massachusetts, USA) using the Fast Real-Time PCR System 7500 (Applied Biosystems, Waltham, Massachusetts, USA). Primer sequences are shown in **Supplementary Table 1** and the average CT values for each gene from three independent experiments are provided in **Supplementary Table 2**. We used the ribosomal protein S16 (*S16)* or hypoxanthine phosphoribosyltransferase (*Hprt*) as reference genes, as indicated in the respective figure legends. To determine the relative change in gene expression of treated samples relative to untreated controls, we calculated the mean cycle threshold (Ct) value from triplicate samples of each condition for each gene, and the 2^-ΔΔCt^ value was determined by the following formula: 2^-ΔΔCt^ = 2 ^– Treated Sample (Mean Ct value Gene of interest –Mean Ct value Reference gene) – Control Samples(Mean Ct value Gene of interest –Mean Ct value Reference gene)^.

### Primary Mouse Brain Microvascular Endothelial Cells Culture

BBB endothelial cells were isolated from the cortex of six to twelve weeks old C57BL/6J male mice according to a previously established protocol (19). After isolation, the cells from one brain were plated in two Matrigel- (Corning, New York, USA, reference 356231) coated 35mm dishes (ibidi GmbH, Munich, Germany) on a surface of 6.6 mm^2^ for live cell imaging experiments or in three filters of 0.5μm pore size and 6mm diameter (3421, Corning, New York, USA), coated with laminin from Engelbreth-Holm-Swarm murine sarcoma basement membrane (Sigma-Aldrich, St. Louis, MO, USA) and Matrigel for migration assays. The cells were grown for seven days at 37°C, 10% CO_2_ in culture media containing Dulbecco´s modified eagle medium (DMEM, Gibco, Paisley, UK) supplemented with 20% FBS (Biowest, Nuaille, France), 2% sodium pyruvate (11360-039, Gibco, Paisley, UK), 2% MEM non-essential amino acids (11140-035, MEM NEAA, Gibco, Paisley, UK), 50µg/ml gentamycin (15710-049, Gibco, Paisley, UK) and 1ng/ml basic fibroblast growth factor (F0291, Sigma-Aldrich, St. Louis, MO, USA). For the first 48h, the media was supplemented with 4µg/ml puromycin (P9620, Sigma-Aldrich, St. Louis, MO, USA) to prevent pericyte contamination. At culture day six, cells were stimulated for 14h-20h with 20ng/ml recombinant murine IL-1β (211-11B-10UG, PeproTech, Rocky Hill, NJ, USA) or with 5ng/ml recombinant murine TNF-α (211-11B-10UG, PeproTech, Rocky Hill, NJ, USA) + 100IU/ml IFN-γ (315-05, PeproTech, Rocky Hill, NJ, USA).

### Primary Mouse Choroid Plexus Epithelial Cells Culture

ChP epithelial cells were isolated, cultured and stimulated according to a previously established protocol (20) with minor adjustments. Specifically, six to twelve weeks old C57BL/6J male mice were sacrificed and the ChP from the lateral and fourth ventricles isolated using a stereomicroscope. Following 30 min digestion at 37°C in 1x DPBS (Gibco, Paisley, UK) containing 0.1mg/ml pronase (Roche Mannheim, Germany), epithelial cells were mechanically and enzymatically disaggregated from the choroidal structure using warm 0.025% trypsin-EDTA (Gibco, Paisley, UK) containing 12.5µg/ml DNAse I (Roche, Mannheim, Germany). After stopping the enzymatic reaction, the cell suspension was resuspended in ChP epithelial cells media containing DMEM/F12 1:1 (Gibco, Paisley, UK), FBS 10% (Gibco, Paisley, UK), 2mM glutamine (Gibco, Paisley, UK), 50µg/ml gentamycin (Gibco, Paisley, UK) and plated for 2h in non-coated 35mm petri dishes (PD) (BD Biosciences, Franklin Lakes, NJ, USA) at 37°C. This step allowed the detachment of fibroblast and macrophages from epithelial cells. The cells were resuspended in ChP epithelial cells media and were plated on 50µg/ml laminin (Roche, Mannheim, Germany) coated inverted filters of 5μm pore size and 6mm diameter (Corning, New York, USA, reference 3421) for 48h, following which the filters were placed in a 24well plate (Thermo Fisher Scientific, Rochester, NY, USA). To obtain a single monolayer, ChP epithelial cells media supplemented with 5μg/ml human insulin (Sigma Aldrich, St Louis, MO, USA), 10ng/ml hEGF (Peprotech, Rocky Hill, NJ, USA), 2μg/ml hydrocortisone (Sigma, Buchs, Switzerland) and 20μm cytosine arabinoside (Ara-C; Sigma, St. Louis, MO, USA) was placed below the filter, in contact with the cells. The apical side of the insert remained dry to prevent the formation of double layer culture. At culture day six, epithelial cells are stimulated whether with 10ng/ml TNF-α (PromoCell, GmbH, Heidelberg, Germany) or with 100IU/ml IFN-γ (PeproTech, Rocky Hill, NJ, USA) for 16h. Unstimulated epithelial cells were used as control conditions.

### Live Cell Imaging Migration Experiment

*In vitro* live cell imaging of MoC interaction with BBB endothelial cells was performed as previously described (21). M^unpolarized^, M^LPS+IFN-γ^ and M^IL-4+IL-13^ macrophages were resuspended in migration assay media (MAM) containing DMEM, 5% FBS, 4mM L-Glutamine (A2916801, Gibco, Paisley, UK) and 25mM HEPES buffer solution (15630-056, Gibco, Paisley, UK) at a concentration of 1 x 10^6^ cells/ml. A total of 2 x 10^5^ cells were used per movie. Accumulation of M^unpolarized^, M^LPS+IFN-γ^ and M^IL-4+IL-13^ macrophages on BBB endothelial cells in the flow chamber occurred for an interval of 5 min, at a low shear pressure of 0.1 dyn/cm^2^, followed by an increase in the shear flow at physiological levels of 1.5 dyn/cm^2^ for 25 min. The total recording time was 30 min, with 10 seconds interval between each frame. Image acquisition was performed using the phase contrast at an inverted microscope (AxioObserver, Zeiss, Feldbach, Switzerland) with a 10x objective. The image analysis was performed using Fiji (National Institute of Health, Bethesda, MD, USA). The number of arrested macrophages per condition was assessed at 40 seconds after onset of physiological shear flow.

### Two-Chamber Migration Assays

To assess MoC migration across BBB endothelial cells or ChP epithelial cells monolayers under static condition, we used a transwell system as previously described (20, 22). BBB endothelial cells and ChP epithelial cells cultured on filters as described above were used at culture day seven after 16h cytokine stimulation, whereas MoCs were used after 48h cytokine stimulation. Following trypsinization from culture plates, MoCs were labelled with 1μm CellTracker™ green dye (CMFDA, C2925, Invitrogen, Rockford, IL, USA) at 37°C for 30 min. After labelling, MoCs were washed twice with 1x DPBS and resuspended in MAM. For each condition, 2x10^5^ MoCs resuspended in 100μl MAM were added on the upper side of the filter. Underneath the filter, 600μl MAM were added. Laminin-coated empty filters were used as controls. MoCs were allowed to migrate across BBB endothelial cells, ChP epithelial cells or across matrigel or laminin coated empty filters for a period of 8h at 37°C, 10% CO_2_. At the end of the experiment, migrated MoCs were collected from the bottom compartment and CMFDA^+^ cells and counted using an Attune NxT flow cytometer (Thermo Fisher Scientific, Rochester, NY, USA). Later, the apical and basolateral sides of the filters were gently washed three times with 1xDPBS and the filters were 1% PFA fixed and stained with polyclonal rabbit anti-zona occludens-1 (ZO-1) antibody (61-7300, Invitrogen, Rockford, IL, USA) to distinguish endothelial monolayer, or with monoclonal mouse anti-human E-cadherin (610182, BD Biosciences, Franklin Lakes, NJ, USA), to delineate the epithelial layer (see below). Following fixation and staining, using a confocal microscope (LSM800 Zeiss, Germany), we acquired 20μm z-stack images starting from the upper side of each filter, with a 2μm interval. For each filter, we acquired the z-stack images at five different fields of view (FOV) (upper left, upper right, center, lower left, lower right), to sample the entire filter area. After image acquisition, using Fiji software, we quantified the number of MoCs attached on the upper side of the filters (corresponding to the luminal side of BBB endothelial cells, and to the basolateral side of inverted ChP epithelial cells), using a custom-made macro (**Supplementary Data 1**). We confirmed correct macro function by manual quantification of at least 30 fields of view from different filters. The quantification of MoCs undergoing migration across monolayers and filters (on the abluminal side of BBB endothelial cells, or on the apical side of ChP epithelial cells) was performed manually. For each filter, the mean and the standard error of the mean (SEM) of the number of BMDMs from five different FOVs was used for final quantification.

### *In Vitro* Interaction of MoCs With BBB Endothelial and ChP Epithelial Cells and G-CSF + GM-CSF Blocking Experiments

MoCs were isolated and differentiated for 8 days in the presence of m-CSF (as described above). BBB endothelial cells and ChP epithelial cells were isolated as described and grown for 7 days in 24 well plates, at a density of 2 brains/well for BBB endothelial cells or 3.3 brains/well for ChP epithelial cells. At culture day six, BBB endothelial cells were stimulated for 14h-16h with 20ng/ml recombinant murine IL-1β (PeproTech, Rocky Hill, NJ, USA, catalog number 211-11B-10UG) or with a mix of 5ng/ml recombinant murine TNF-α (PeproTech, Rocky Hill, NJ, USA, catalog number 211-11B-10UG) + 100IU/ml IFN-γ (PeproTech, Rocky Hill, NJ, USA, catalog number 315-05). In contrast, ChP epithelial cells were stimulated whether with 10ng/ml TNF-α (PromoCell, GmbH, Heidelberg, Germany) or with 100IU/ml IFN-γ (PeproTech, Rocky Hill, NJ, USA). Unstimulated BBB endothelial cells/ChP epithelial cells were used as control conditions.

Before the interaction assay and following BBB endothelial cells and ChP epithelial cells cytokine activation, the CNS barrier models were washed thoroughly with 1xPBS (Gibco, Paisley, UK) and MoCs were collected by 10min incubation in 0.05% Trypsin (Gibco, Paisley, UK, reference 25300-054) at 37°C. A total of 1.2 x 10^6^ MoCs were resuspended in 500µl migration assay media (DMEM, 5% FBS, 4mM L-Glutamine, 25mM HEPES, 50µg/ml gentamycin) and added to BBB endothelial cells or ChP epithelial cells containing wells.

For functional assays investigating the roles of factors released by CNS *in vitro* barriers on macrophages polarization, prior to the addition of MoCs, BBB endothelial cells/ChP epithelial cells were incubated for 1h30-2h with neutralizing antibodies against G-CSF (polyclonal goat IgG, AF-414-NA, R&D Systems), GM-CSF (polyclonal goat IgG, AF-415-NA, R&D Systems), or with isotype control (polyclonal normal goat IgG, AB-108-C, R&D Systems) at a concentration of 10µg/ml per antibody. The following mix of antibodies was used: 10µg/ml G-CSF + 10µg/ml GM-CSF. The antibody mixes were further kept in the culture throughout the 7h incubation of MoCs with BBB endothelial/ChP epithelial cells.

Following the 7h incubation, MoCs were recovered from the BBB endothelial cells/ChP epithelial cells containing wells *via* 3-4 rounds of resuspensions. Following 2x washing steps of both BBB endothelial cells/ChP epithelial cells and MoCs, the cells were further processed for mRNA isolation, and cDNA synthesis. Lastly, we assessed the expression of *Arg1, Tpi1, Gpi1* genes in MoCs by means of RT-qPCR.

### Incubation of MoCs With Recombinant Proteins

Following isolation, culture and differentiation for 8 days, MoCs were trypsinized, plated in 24-well plates at a concentration of 1.2x10^6^ and incubated for 7h with 10ng/ml, 50ng/ml or 100ng/ml of recombinant mouse GM-CSF or G-CSF proteins, at 37°C, 10% CO_2._ At the end of the experiment, the attached BMDMs were washed 3x with 1xPBS and were further processed for mRNA isolation, cDNA synthesis and RT-qPCR.

### Immunofluorescence Staining of *In Vitro* CNS Barrier Cells Cultured on Filters

BBB endothelial cells and ChP epithelial cells cultured on Transwell filters (Corning, New York, USA, 3421) were fixed with 1% PFA diluted in 1x DPBS for 10min at RT. After fixation and removal from the inserts, the filters were washed three times with 1x DPBS and incubated in blocking buffer containing 5% skimmed milk (Rapilait, Migros, Switzerland), 0.3% Triton-X-100 (Sigma-Aldrich, St Louis, MO, USA), 0.04% NaN_3_ (Fluka Chemie, Buchs, Switzerland), pH 7.4 for 30min at RT. The cells were incubated afterwards in primary antibodies for 1h at RT. The following primary antibodies were used for immunofluorescence staining: polyclonal rabbit anti-ZO-1 antibody (Invitrogen, Rockford, IL, USA, catalog number 61-7300), monoclonal mouse anti-β-catenin (BD Biosciences, Franklin Lakes, NJ, USA, catalog number 610154), monoclonal mouse E-cadherin (BD Biosciences, Franklin Lakes, NJ, USA, catalog number 610182), polyclonal rabbit anti-claudin-5 (Thermo Fisher Scientific, Rockford, IL, USA, catalog number 341600), monoclonal rat CD106 (clone 429 MVCAM.A, BD Biosciences, 553329). Rat anti-mouse endothelial-selectin (E-Selectin, clone 10E9), rat anti-mouse intracellular adhesion molecule-1 (ICAM-1, clone 25ZC7), rat anti-mouse vascular cell adhesion molecule-1 (VCAM-1, clone 9DB3), rat anti-mouse vascular endothelial cadherin (VE-cadherin, clone 11D4) and rat anti-mouse junctional adhesion molecule-A (JAM-A, clone BV12) antibodies were isolated from the supernatants of hybridoma cultures in house. After 3x DBPS washing steps, the samples were incubated in secondary antibodies diluted in blocking buffer for 1h at RT, under light protected conditions. The following secondary antibodies were used: AF488 donkey anti-mouse IgG (H+L) (Invitrogen, Eugene, OR, USA, catalog number A21202), AF647 goat anti-rabbit IgG (H+L) (Invitrogen, Eugene, OR, USA, catalog number A21244), Cy3-conjugated AffiniPure donkey anti-rat IgG (H+L) (Jackson ImmunoResearch, West Grove, Pa, USA, catalog number 712-165-150). Following nuclear staining with DAPI (1:5000, stock concentration of 1mg/ml, AppliChem, Darmstadt, Germany) for 5 min at RT, the filters were washed 3x with 1x DPBS, placed on glass slides (Thermo Scientific, Rochester, NY, USA) and mounted with embedding medium Mowiol (Sigma-Aldrich, St Louis, MO, USA).

### Flow Cytometry Analysis of MoC Integrin Expression

After 48h polarization (see above), M^Unpolarized^, M^LPS+IFN-γ^ and M^IL-4+IL-13^ were detached from culture plates using 0.05% trypsin/EDTA solution (Merck, Darmstadt, Germany) for 10 min at 37°C. After stopping the reaction with MoC media, cells were washed with 1x DPBS and the Fc-receptors were blocked on ice for 15 min (using anti-CD16/32, homemade solution). The cells were then incubated with the following antibodies diluted in 1x DPBS for 30 min at 4°C, in light-protected conditions: PE-Cy7 conjugated anti-mouse CD11b (clone M1/70, BioLegend, San Diego, CA, USA, catalog number 101216); BV711 conjugated anti-mouse CD45 (clone 30-F11, Biolegend, San Diego, CA, USA, catalog number 103147); fluorescein isothiocyanate (FITC) conjugated anti-mouse CD18 (β2) (clone M18/2, Invitrogen, Rockford, IL, USA, catalog number 11-0181-82); APC-efluor 780-conjugated anti-mouse CD29 (β1) (clone HMb1-1, Invitrogen, Rockford, IL, USA, catalog number 47-0291-82), alexa fluor 647-conjugated anti-mouse CD49d (α4) (BioRad, Hercules, CA, USA, catalog number MCA 1230A647T), cell viability dye eFluor 506 (Invitrogen, Rockford, IL, USA, catalog number 65-0866-14). Isotype control stainings served as controls. Samples were acquired using an Attune NxT cytometer (Thermo Fisher Scientific, Rochester, NY, USA). M^Unpolarized^, M^LPS+IFN-γ^ and M^IL-4+IL-13^ were gated according to their forward- and side-scattering, viability and CD11b^+^CD45^+^ expression. Analysis was performed using the FlowJo™ software (version 10, Ashland, OR, USA) and the relative mean fluorescence intensity (MFI) was calculated for each antibody by subtracting the MFI of antibody staining from the MFI of isotype control staining.

### Statistics

Statistical analysis was performed using GraphPad Prism 8 or 9 software (La Jolla, CA, USA). All values are presented as mean ± SEM. Asterisks indicate significant differences (∗p < 0.05, ∗∗p < 0.01 and ∗∗∗p < 0.001, ∗∗∗∗p < 0.0001). Unpaired T test was used for the analysis of M^iNOS^, M^Arginase^ and M^iNOS/Arginase^ cells in the IL1R1^+^ and IL1R1^negative^ vessel regions and for the analysis of the percentage of Il1R1^positive^ vessels in the parenchyma and the meninges following validation of their normal distribution (by Shapiro Wilk and Kolmogorov-Smirnov tests). One-way ANOVA with Tukey’s multiple comparison test was used for the following experiments: TEER measurements of BBB endothelial cells and ChP epithelial cells, MoC integrin expression by flow cytometry, Mean Fluorescence Intensity assessment of VCAM-1, ICAM-1 and E-selectin expression on BBB endothelial and ChP epithelial cells. MoC mRNA expression of inflammatory and chemokine receptors genes, quantification of CCR2^+^ and CX3CR1^+^CCR2^negative^ cells in the ChP, quantification of M^iNOS^, M^Arginase^ and M^iNOS/Arginase^ cells in the perivascular and meningeal space, MoC incubation with recombinant G-CSF, GM-CSF proteins, MoC incubation with BBB endothelial cells and with ChP epithelial cells, MoC incubation with BBB endothelial cells/ChP epithelial cells in the presence of G-CSF+GM-CSF blocking antibodies, mRNA expression of *Csf2, Csf3* in unstimulated and cytokine activated BBB endothelial cells and ChP epithelial cells. Two-way ANOVA with Tukey’s multiple comparison tests was used to assess statistical significance in MoC migration assays with ChP epithelial cells and BBB endothelial cells, both in presence and absence of physiological shear flow.

## Results

### Differential Distribution of Polarized MoCs at CNS Barriers During EAE

Upon autoimmune inflammation, CNS interfaces become increasingly populated by tissue-invading MoCs (23, 24), some of which display a complete functional polarization characterized by the expression of iNOS and/or arginase-1 (17). Whether M^iNOS^ and M^Arginase^ MoCs accumulate equally at different CNS gateways remains however unknown.

**Figure 1:**
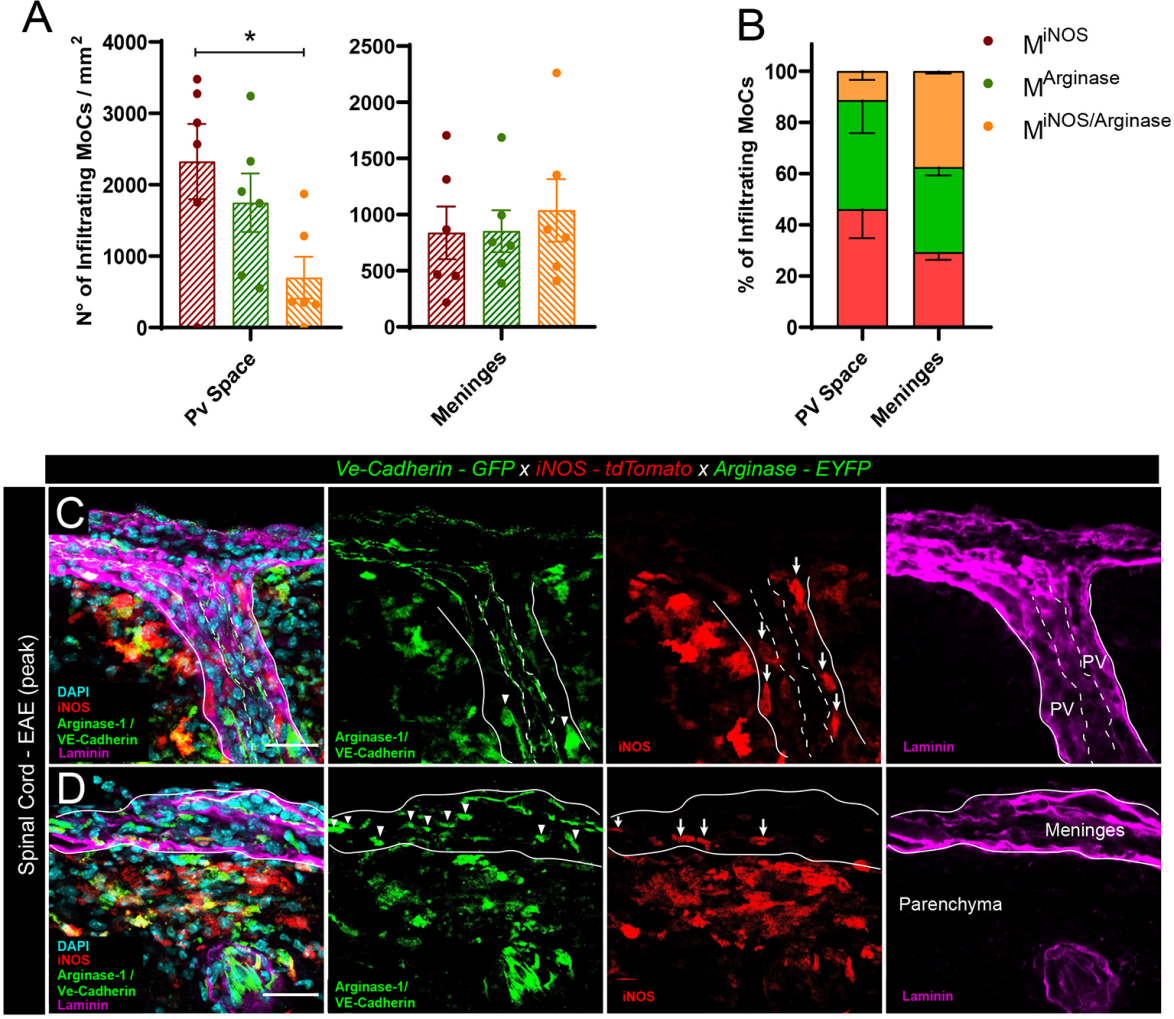
Polarized MoC density in perivascular and leptomeningeal spaces during EAE. **(A)** Density of M^iNOS^, M^Arginase^ and M^iNOS/Arginase^ cells in the perivascular spaces and leptomeninges of *VE-cadherin-GFP x iNOS-tdTomato x Arginase-EYFP* mice induced with EAE (6 mice at day 3 after onset of the disease). Data represented as mean ± SEM. Statistical analysis performed by one-way ANOVA with Tukey’s multiple comparisons test, p=0.0389, *p < 0.05. **(B)** Relative percentage of the M^iNOS^, M^Arginase^ and M^iNOS/Arginase^ cells shown in **(A)**. Within perivascular spaces, a higher percentage of M^iNOS^ cells are detected (Mean = 46.09%, SEM = 11.34) as compared to M^Arginase^ (Mean = 42.47%, SEM = 12.80) and M^iNOS/Arginase^ (Mean = 11.44%, SEM = 3.46). In contrast, within the meninges, a predominant population of M^Arginase^ (Mean = 33.24%, SEM = 3.23) and M^iNOS/Arginase^ (Mean = 37.49%, SEM = 0.97) was found, as compared to M^iNOS^ (Mean = 29.27%, SEM = 2.91). No statistically significant differences were observed, one-way ANOVA with Tukey´s multiple comparisons test. **(C, D)** Representative confocal images of spinal cord perivascular spaces **(C)** and leptomeninges **(D)** of *VE-cadherin-GFP x iNOS-tdTomato x Arginase-EYFP* mice induced with EAE (3 days after disease onset). tdTomato indicates M^iNOS^, EYFP/GFP M^Arginase^ cells and VE-cadherin of endothelial cell junctions. Staining with DAPI reveals nuclei, immunostaining with anti-laminin antibody reveals the perivascular and meningeal extracellular matrix. Arrowheads highlight M^Arginase^ cells and arrows indicate M^iNOS^ cells in the perivascular space **(C)** and meninges **(D)**. Scale bar, 30 μm.

To quantify the presence of polarized MoCs at the distinct CNS barriers we created triple transgenic *VE-cadherin-GFP x iNOS-tdTomato x Arginase-EYFP* mice, a model in which VE-cadherin^+^ endothelial junctions are visualized by GFP expression whereas pro-inflammatory M^iNOS^ and anti-inflammatory M^Arginase^ cells are visualized by tdTomato and EYFP expression, respectively (25). In addition, we performed pan-laminin staining of the tissue sections allowing us to identify endothelial and parenchymal basement membranes and thus the actual borders of the perivascular and subarachnoid spaces, respectively. Our analysis revealed that, upon induction of EAE, CNS vessels displayed preferential perivascular accumulation of M^iNOS^ cells, which occupied these spaces in significant higher numbers than transitional M^iNOS/Arginase^ cells (**Figures 1A–C**). Conversely, leptomeningeal spaces revealed an equal presence of M^iNOS^, M^Arginase^ and M^iNOS/Arginase^ cells (**Figures 1A, B, D**).

**Figure 2:**
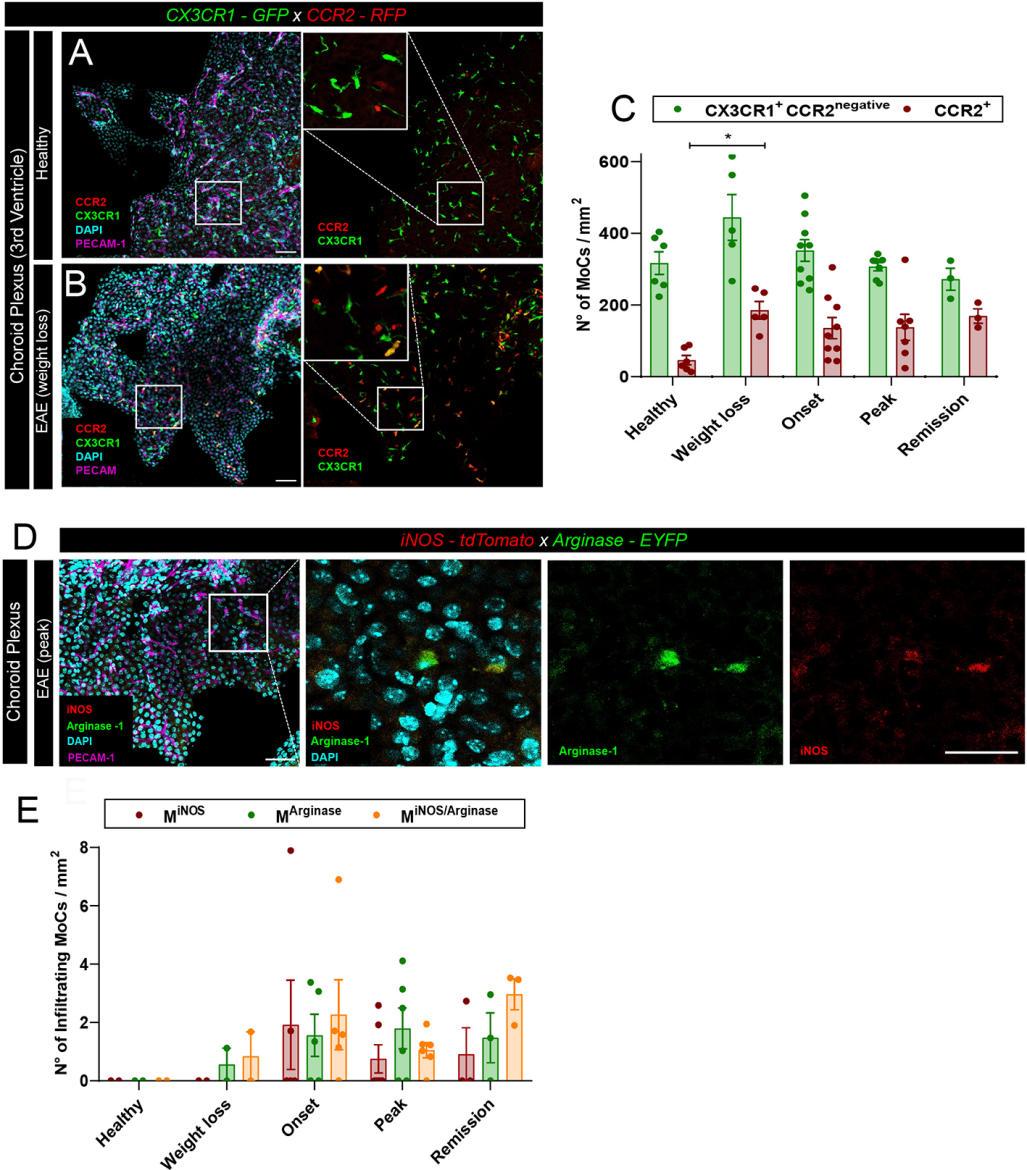
MoC accumulation in the ChP during EAE. **(A, B)** Representative confocal images of the ChP from the third ventricle of **(A)** a healthy *CX3CR1-GFP x CCR2-RFP* mouse and **(B)** from a *CX3CR1-GFP x CCR2-RFP* mouse induced with EAE (weight loss stage). RFP^+^ and GFP^+^ cells indicate the presence of CCR2^+^ MoCs and CX3CR1^+^ tissue resident cells respectively. Staining with DAPI reveals nuclei, PECAM-1-specific immunostaining reveals endothelial cells. Scale bar, 50 μm; magnified regions, scale bar 30 μm. **(C)** Density of RFP^+^ (CCR2^+^) and GFP^+^ RFP^negative^ cells (CX3CR1^+^CCR2^negative^) from the ChP of the third, fourth and lateral ventricles of *CX3CR1-GFP x CCR2-RFP* mice at different EAE disease stages post immunization. Analysis includes 6 healthy control mice, 5 mice at preclinical weight loss, 9 mice at clinical onset, 7 mice at symptomatic peak and 3 mice at clinical remission. Data is represented as mean ± SEM, one-way ANOVA with Tukey´s multiple comparison test. A statistically significant increase in CCR2^+^ cell density in the ChP was observed at weight loss, compared to a healthy status, *p* = 0.0463, *p < 0.05. **(D)** Representative confocal images of the ChP from the third ventricle of an *iNOS-tdTomato x Arginase-EYFP* mouse induced with EAE (symptomatic peak). tdTomato indicates M^iNOS^, EYFP M^Arginase^ cells. Staining with DAPI reveals nuclei, immunostaining with PECAM-1-specific antibodies reveals endothelial cells. Scale bar, 50 μm; magnified regions, scale bar 30 μm. **(E)** Density of M^iNOS^, M^Arginase^ and M^iNOS/Arginase^ cells from the ChP of *iNOS-tdTomato x Arginase-EYFP* mice at different disease stages (analysis includes 2 healthy control mice, 2 mice at weight loss, 5 mice at clinical onset, 6 mice at symptomatic peak, 3 mice at disease remission). Data is represented as mean ± SEM.

Leptomeningeal MoC accumulation can derive from local extravasation of monocytes through meningeal vessels or from CSF trafficking through the distal ChP (24). To visualize the density of MoCs at the ChP, we first made use of *CX3CR1-GFP x CCR2-RFP* mice, a model in which differential reporter expression allows distinction of infiltrating CCR2^+^ MoCs from long-lived CCR2^negative^CX3CR1^high^ resident macrophages (9). In healthy animals, confocal analysis of thick ChP sections revealed a minor presence of blood borne CCR2^+^ MoCs (26) and a high number of ramified CX3CR1^+^ tissue resident macrophages populating the ChP stroma (**Figure 2A**). During EAE development, we could observe a significant increase in CCR2^+^ infiltrating cells in the ChP stroma (**Figures 2B, C**), suggesting that this region allows CNS infiltration of MoCs during autoimmune inflammation.

To assess the functional polarization of these CCR2^+^ MoCs, we induced EAE in *iNOS-tdTomato x Arginase-EYFP* mice and performed confocal analysis of thick brain sections, observing equal presence of M^iNOS^, M^Arginase^ and M^iNOS/Arginase^ cells in the ChP stroma (**Figures 2D, E**). Nonetheless, compared to the high cellular density observed at the other CNS barriers (**Figure 1A**), only few polarized MoCs could be observed within the ChP throughout disease development (**Figure 2E**).

Taken together, our data indicate that pro- and anti-inflammatory MoCs are present at all CNS border areas during EAE. However, these cells accumulate in different densities and proportions at distinct CNS barriers, potentially indicating preferential trafficking routes and diverse local molecular activation of migrating MoCs.

### Functional Characterization of Primary CNS Barrier and MoC *In Vitro* Models

To study the differential dynamics of MoCs at the distinct CNS barriers, we adopted an *in vitro* system comprising bone marrow-derived macrophage cultures [to mimic MoCs), primary mouse brain microvascular endothelial cells (to mimic the BBB endothelial cells (19, 27)] and primary mouse ChP epithelial cells [to mimic the blood-CSF barrier (20)], all isolated according to well-established approaches.

BBB endothelial cells formed a confluent monolayer, characterized by junctional localization of the adherens junction proteins β-catenin and VE-cadherin and of the tight junction proteins zona occludens-1 (ZO-1) and claudin-5 (**Supplementary Figures 1A–D**). To model neuroinflammatory conditions, BBB endothelial cells were stimulated either with interleukin-1β (IL-1β) or with tumor necrosis factor-α (TNF-α) + interferon-γ (IFN-γ) (27, 28). Cell surface protein expression of the integrin-binding molecule intercellular adhesion molecule-1 (ICAM-1) was specifically upregulated upon IL-1β stimulation as was the endothelial cell adhesion molecule E-selectin, while expression of vascular cell adhesion molecule-1 (VCAM-1) was not affected upon cytokine stimulation compared to unstimulated BBB endothelial cells (**Supplementary Figure 1E–J**). As a measure of barrier integrity, we assessed trans-endothelial electrical resistance (TEER) of the BBB endothelial cells and observed a significant decrease of TEER following cytokine stimulation compared to unstimulated conditions (**Supplementary Figure 1K**).

To allow for side-by-side comparison of the BBB with the blood-CSF barrier, ChP epithelial cells were isolated from the same mice that yielded BBB endothelial cells. ChP epithelial cells displayed mature BSCFB characteristics as shown by the junctional localization of E-cadherin, junctional adhesion molecule-A (JAM-A) and β-catenin (**Supplementary Figure 2A–C**). To mimic local inflammation, ChP epithelial cells were stimulated either with TNF-α or IFN-γ (20). IFN-γ stimulation led to upregulation of ICAM-1 and VCAM-1 (**Supplementary Figures 2D–G**). However, we found no increase in TEER across cytokine-stimulated ChP epithelial cells (**Supplemetary Figure 2H**).

To model MoCs trafficking through CNS barriers, we made use of bone marrow-derived macrophage cultures and implemented a stimulus-based nomenclature according to suggestions by experts in the field (29). We kept cells unstimulated (M^unpolarized^) or polarized them towards a pro- (M^LPS+IFN-y^) or anti-inflammatory phenotype (M^IL-4+IL-13^), observing significant upregulation of pro-inflammatory genes including *Nos2* and anti-inflammatory genes including *Arg1*, respectively (**Supplementary Figures 3A, B** and **Supplementary Table 2**) (17).

Secondly, we assessed the expression of chemokine receptors that might affect cell migration. We observed an equal expression of *Ccr5* in all conditions, an increase of *Ccr1* expression in M^LPS+IFN-y^ and M^IL-4+IL-13^ compared to M^unpolarized^ and a significant decrease of *Ccr2* expression in M^LPS+IFN-y^ cells compared to M^unpolarized^ and M^IL-4+IL-13^ cells (**Supplementary Figure 3C** and **Supplementary Table 2**). Signaling through chemokine receptors also contributes to integrin activation (30) in a phenotype-dependent manner (31, 32). We thus characterized surface expression of key integrins required for MoC interaction with CNS barrier cells (30, 33, 34) and observed high basal expression of β2, α4 and β1 integrin subunits in all MoCs, with β2 integrin significantly more expressed in M^IL-4+IL-13^ cells (35) (**Supplementary Figure 3D**).

In conclusion, we tested *in vitro* models of the BBB and of the blood-CSF barrier which show mature barrier characteristics. In parallel, we could observe that differential MoC polarization significantly affects expression of key molecules involved in cell dynamics, which may contribute to the different interaction of MoCs with distinct CNS barriers.

### Functional Polarization Decreases MoC Adhesion to the BBB and Migration *In Vitro*

To study the migration of MoCs across CNS barriers, we used a two-chamber transmigration system in which CMFDA-labelled MoCs were co-incubated with BBB endothelial cells.

**Figure 3:**
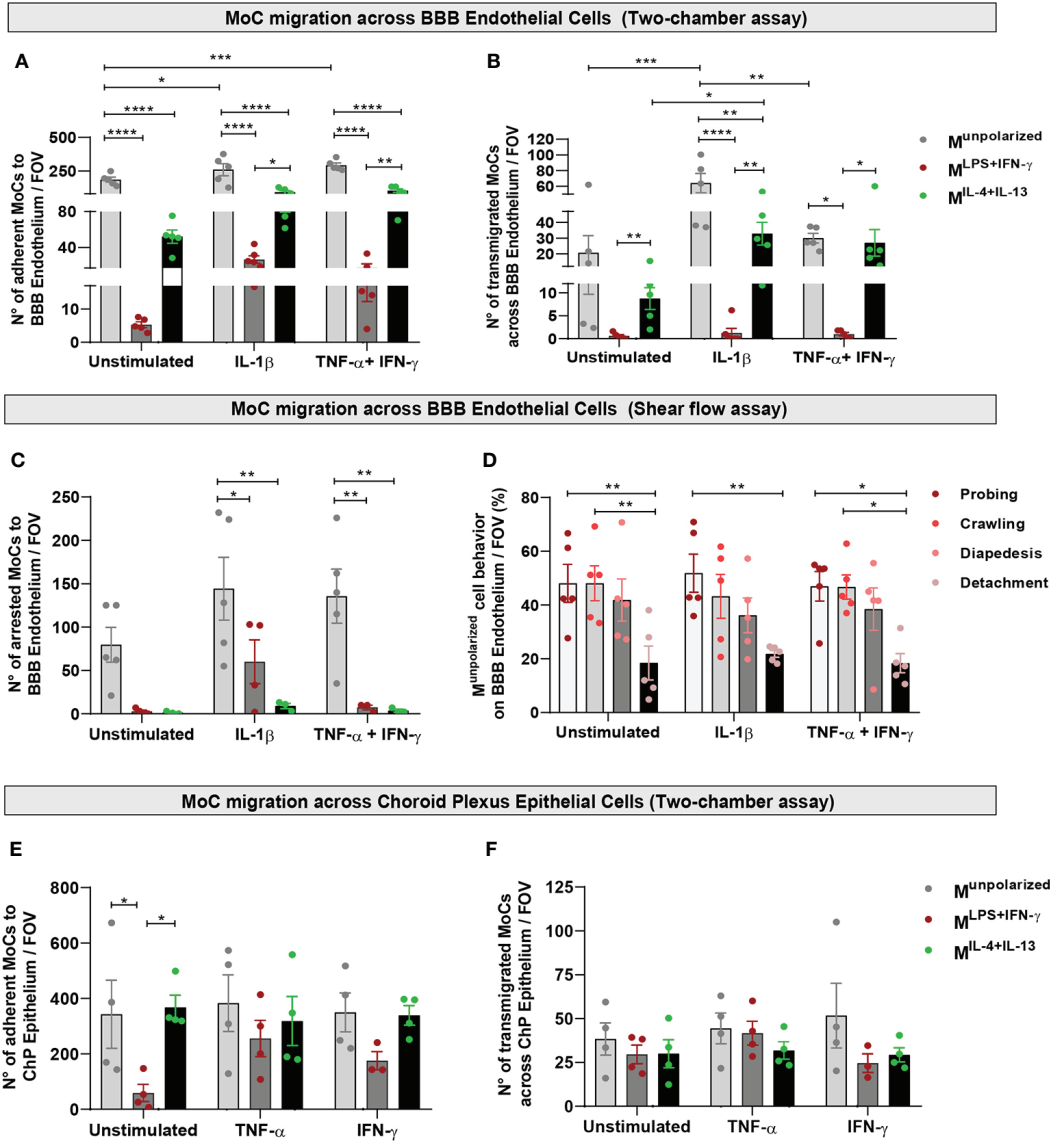
MoC migration across BBB endothelial and ChP epithelial cells *in vitro*. **(A, B)** M^unpolarized^, M^LPS+IFN-γ^ and M^IL-4+Il-13^ cells were allowed to migrate across a monolayer of unstimulated, IL-1β or TNF-α+IFN-γ stimulated BBB endothelial cells in a two-chamber system. **(A)** Number of M^unpolarized^, M^LPS+IFN-γ^ and M^IL-4+Il-13^ cells attached on the luminal side of BBB endothelial cells following 8h incubation. Data points represent mean number of cells per filter, with five fields of view (FOV) analysed per filter, four independent experiments (one experiment was performed in duplicates). Displayed are mean ± SEM, two-way ANOVA with Tukey´s multiple comparison, *p < 0.05, **p < 0.01, ***p < 0.001, ****p < 0.0001. On both unstimulated and cytokine activated BBB endothelial cells, M^unpolarized^ cells attached with higher efficiency compared to both M^LPS+IFN-γ^ and M^IL-4+IL-13^ (M^unpolarized^ vs M^LPS+IFN-γ^ p < 0.0001; M^unpolarized^ vs M^IL-4+IL-13^ p < 0.0001). M^unpolarized^ attached more to IL-1β BBB endothelial cells (p = 0.019) and TNF-α+IFN-γ BBB endothelial cells (p = 0.006) as compared to unstimulated BBB endothelial cells. Compared to M^LPS+IFN-γ^, M^IL-4+IL-13^ cells adhered significantly more to IL-1β (p = 0.033) and TNF-α+IFN-γ stimulated BBB endothelial cells (p = 0.005). **(B)** Number of MoCs which migrated on the abluminal side of BBB endothelial cells after 8h incubation. Data points represent mean number of cells per filter, with five fields of view (FOV) analysed per filter, four independent experiments (one experiment was performed in duplicates). Displayed are mean ± SEM, two-way ANOVA with Tukey´s multiple comparisons test, *p < 0.05, **p < 0.01, ***p < 0.001, ****p < 0.0001. A significantly higher number of M^unpolarized^ cells underwent diapedesis across IL-1β stimulated BBB endothelial cells compared to both M^LPS+IFN-γ^ (p < 0.0001) and M^IL-4+Il-13^ (p = 0.0057) cells. M^unpolarized^ also showed increased migration across TNF-α+IFN-γ BBB endothelial cells compared to M^LPS+IFN-γ^ cells (p < 0.011). Compared to M^LPS+IFN-γ^, M^IL-4+IL-13^ cells migrated in higher numbers across both IL-1β (p = 0.004) and TNF-α+IFN-γ BBB endothelial cells (p = 0.024). Within its group, M^unpolarized^ cells migrated more efficiently through IL-1β compared to both unstimulated (p =0.0001) and TNF-α+IFN-γ BBB endothelial cells (p = 0.003). Polarized MoCs also show different migration efficiencies: M^IL-4+IL-13^ migrate in higher numbers across IL-1β stimulated BBB endothelial cells than across unstimulated endothelium (p = 0.04). Additionally, M^IL-4+IL-13^ migrate in higher numbers across both IL-1β BBB endothelial cells (p = 0.0035) and TNF-α+IFN-γ BBB endothelial cells (p = 0.024) as compared to M^LPS+IFN-γ^ cells. **(C)** M^unpolarized^, M^LPS+IFN-γ^ and M^IL-4+Il-13^ cells were allowed to migrate and interact with unstimulated, IL-1β and TNF-α+IFN-γ stimulated BBB endothelial cells under physiological flow (1.5 dyn/cm^2^) for a period of 25 minutes. Shown is the number of arrested cells on BBB endothelial cells per field of view (FOV). M^unpolarized^ cells displayed superior abilities to adhere to both IL-1β (M^unpolarized^ vs M^LPS+IFN-γ^ p = 0.041; M^unpolarized^ vs M^IL-4+Il-13^ p = 0.002) and TNF-α+IFN-γ stimulated endothelial cells (M^unpolarized^ vs M^LPS+IFN-γ^ p = 0.004; M^unpolarized^ vs M^IL-4+IL-13^ p = 0.003). **(D)** Migratory behaviour of M^unpolarized^ cells on BBB endothelial cells under physiological flow conditions, during 25 minutes live cell imaging. A significantly lower number of cells detached from the endothelium following attachment, compared to the cells that crawled or probed the monolayer (unstimulated BBB endothelial cells: probing vs detachment p = 0.009, crawling vs detachment p = 0.009; IL-1β BBB endothelial cells: probing vs detachment p = 0.008; TNF-α+IFN-γ BBB endothelial cells: probing vs detachment p = 0.013, crawling vs detachment p = 0.014). Displayed are the results from 4 independent experiments, mean ± SEM shown, two-way ANOVA with Tukey´s multiple comparisons test, *p < 0.05, **p < 0.01. **(E, F)** M^unpolarized^, M^LPS+IFN-γ^ and M^IL-4+Il-13^ cells were allowed to migrate across a monolayer of unstimulated, TNF-α or IFN-γ stimulated ChP epithelial cells in a two-chamber system. **(E)** Numbers of M^unpolarized^, M^LPS+IFN-γ^, M^IL-4+IL-13^ adhering on the basolateral side of ChP epithelial cells following 8h incubation. M^unpolarized^ and M^IL-4+IL-13^ display superior abilities to adhere to unstimulated ChP epithelial cells compared to M^LPS+IFN-γ^ cells (M^unpolarized^ *vs* M^LPS+IFN-γ^ p = 0.03; M^IL-4+IL-13^ *vs* M^LPS+IFN-γ^ p = 0.017). Data points represent mean number of cells per filter, with five fields of view (FOV) analysed per filter, four independent experiments. Displayed are mean ± SEM. Two-way ANOVA with Tukey´s multiple comparisons test, *p < 0.05. **(F)** Number of MoCs that migrated toward the apical side of ChP epithelial cells. No statistically significant differences were detected between conditions. Data points represent mean number of cells per filter, with five fields of view (FOV) analysed per filter, four independent experiments. Displayed are mean ± SEM.

Using confocal microscopy, we observed that M^unpolarized^ cells adhered to the luminal side of unstimulated BBB endothelial cells in a significantly higher number compared to polarized MoCs (**Figure 3A**). This also resulted into higher presence of transmigrated M^unpolarized^ on the abluminal BBB endothelial cell side compared to polarized cells (**Figure 3B**). Activation of BBB endothelial cells with the pro-inflammatory cytokines TNF-α+IFN-γ or IL-1β further increased M^unpolarized^ cell attachment and diapedesis (**Figures 3A, B**).

Physiological shear flow within the vascular lumen plays a critical role in immune cell migration (36). To mimic these forces, we used an established live cell imaging approach (21) and allowed MoCs to interact with BBB endothelial cells under physiological flow conditions (1.5 dyn/cm^2^). In accordance to our observations under static conditions, both M^LPS+IFN-y^ and M^IL-4+IL-13^ cells showed significantly reduced adhesion to BBB endothelial cells compared to M^unpolarized^ cells (**Figure 3C**). Following their shear resistant arrest, M^unpolarized^ cells started to probe the BBB monolayer surface or to crawl over the luminal side of BBB endothelial cells, both behaviors eventually leading to diapedesis (**Figure 3D**).

Taken together, pro- or anti-inflammatory MoCs showed reduced abilities to interact with the luminal surface of the BBB, thus suggesting that infiltrating MoCs cannot become fully functionally polarized at the luminal side of the BBB.

### Polarized MoCs Efficiently Interact With the Blood-CSF Barrier Epithelium *In Vitro*

The ChP has been proposed as a key gateway for CNS-invading immune cells (2, 37, 38). CCR2^+^ MoCs can accumulate in the ChP stroma following extravasation across local fenestrated capillaries and could subsequently cross the epithelial blood-CSF barrier to enter the CSF. However, no formal demonstration of this pathway exists in EAE and MS (24).

To mimic MoC dynamics at this CNS gateway, we used a two-chamber transmigration assay allowing CMFDA-stained M^unpolarized^, M^LPS+IFN-y^ and M^IL-4+IL-13^ cells to interact with the basolateral side of ChP epithelial cell monolayers cultured on inverted filters, thus simulating the physiological orientation of epithelial cells within the ChP (20). Interestingly, M^unpolarized^ and M^IL-4+IL-13^ cells adhered in significantly higher numbers to the basolateral side of unstimulated ChP epithelial cells compared to M^LPS+IFN-y^ cells, while cytokine-stimulated ChP epithelial cells allowed efficient adhesion of all M^unpolarized^, M^LPS+IFN-y^ and M^IL-4+IL-13^ cells (**Figure 3E**). Also, a comparable absolute number of M^LPS+IFN-y^, M^IL-4+IL-13^ and M^unpolarized^ cells could move across the epithelial ChP epithelial cell monolayer (**Figure 3F**).

Taken together, our *in vitro* observations suggest that the ChP might constitute a permissive gateway potentially allowing CSF access to MoCs.

### Differential Activation of CNS Barrier Cells Primes MoCs Toward Distinct Functional Fates

The accumulation of polarized MoCs at CNS barriers following EAE induction suggests that invading MoCs can acquire a pro- or anti-inflammatory phenotype early during the invasion process. While a series of transcriptional and morphological changes occurring in monocytes interacting with endothelial cells have been described (39, 40), whether MoCs can acquire a specific functional state during their interaction with CNS barrier cells remains unclear.

To address this question, we analyzed the transcriptional profile of M^unpolarized^ cells incubated with BBB endothelial and ChP epithelial cells activated by different inflammatory stimuli present during EAE (41). Transcriptional analysis of barrier-interacting MoCs revealed enhanced expression of genes encoding for proteins participating in glycolysis, indicating an increased metabolic activation (**Supplementary Figure 4A–D** and **Supplementary Table 3**). A small increase in *Il6* and *Il6r* expression was observed in M^unpolarized^ cells incubated with both BBB endothelial and ChP epithelial cells (**Supplementary Figure 4E, F** **)**; similarly, an increased (albeit not significantly) *Mmp2* expression was detected in MoCs upon incubation with both barriers (**Supplementary Figure 4G, H** and **Supplementary Table 3**). *Il1b* showed a tendency toward upregulation following M^unpolarized^ incubation with ChP epithelial cells and a significantly higher expression in MoCs interacting with IL-1β-stimulated BBB endothelial cells (**Supplementary Figure 4G, H** and **Supplementary Table 3**). Thus, interaction with CNS barrier cells triggered a general cellular activation in M^unpolarized^ cells.

**Figure 4:**
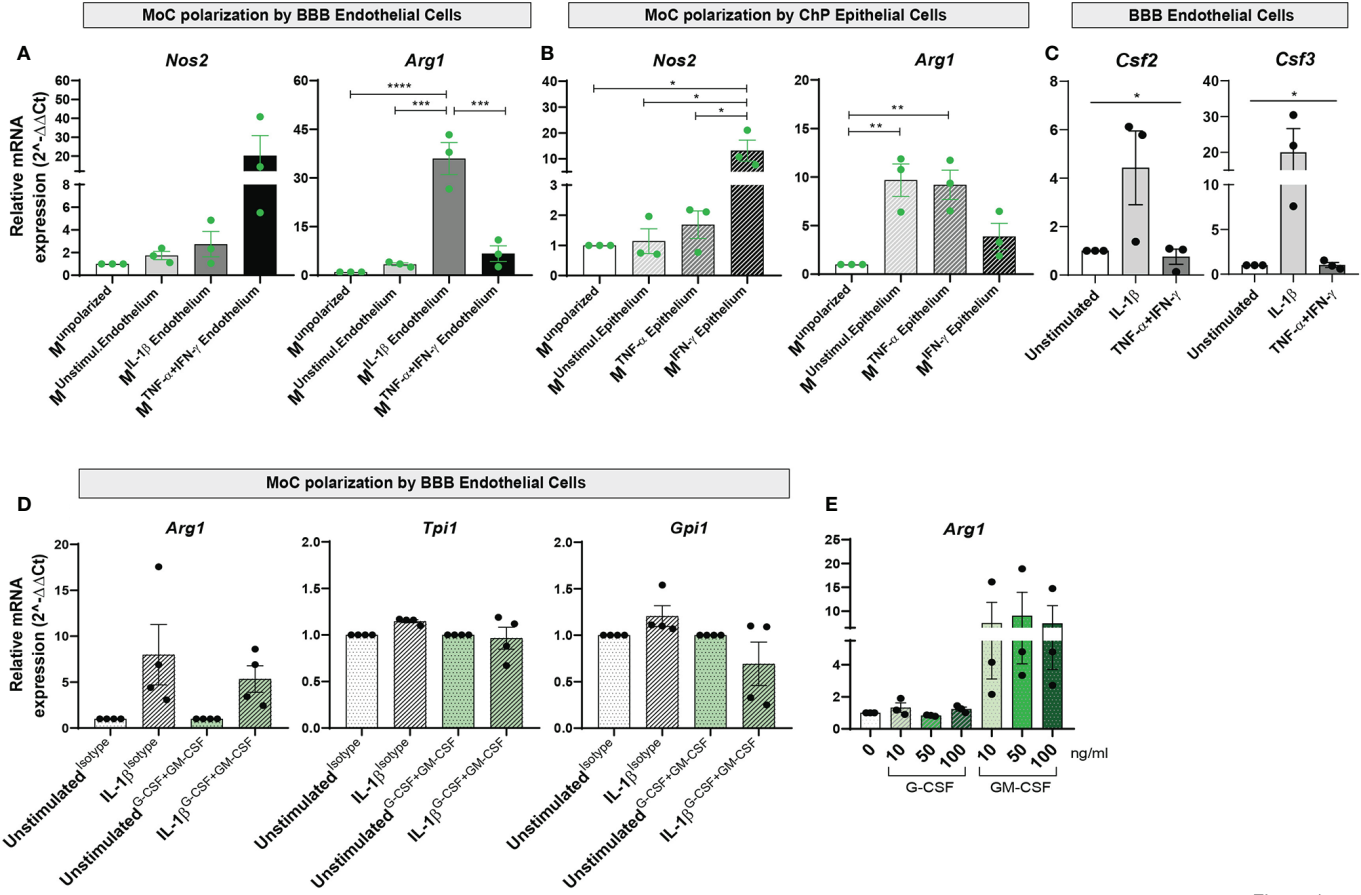
Interaction with BBB endothelial and ChP epithelial cells drives functional specification in MoCs. Relative mRNA expression of *Nos2* and *Arg1* genes in M^unpolarized^ MoCs following 7h incubation with **(A)** BBB endothelial cells and **(B)** ChP epithelial cells. **(A)** Incubation with TNF-α+IFN-γ BBB endothelial cells led to *Nos2* upregulation in MoCs (M^unstimul.Endothelium^ Mean = 1.74, SEM = 0.37; M^IL-1β Endothelium^ Mean = 2.74, SEM = 1.12; M^TNF-α+IFN-γ Endothelium^ Mean = 20.26, SEM = 10.6). In contrast, *Arg1* relative mRNA expression was significantly increased in MoCs incubated with IL-1β BBB endothelial cells (M^IL-1β Endothelium^) as compared to M^unpolarized^ cells (p < 0.0001), to M^unstimulated Endothelium^ (p = 0.0001) and M^TNF-α+IFN-γ Endothelium^ (p = 0.0003). **(B)** MoCs incubated with IFN-γ stimulated ChP epithelial cells (M^IFN-γ Epithelium^) significantly upregulated *Nos2* relative mRNA expression compared to M^unpolarized^ (p = 0.013), to M^unstimulated Epithelium^ (p = 0.014) and to M^TNF-α Epithelium^ (p = 0.018). Similarly, MoCs incubated with unstimulated ChP epithelial cells (M^unstimulated Epithelium)^ (p = 0.007) or with TNF-α ChP epithelial cells (M^TNF-α Epithelium^) (p = 0.01) displayed increased *Arg1* mRNA expression. **(C)** mRNA expression of *Csf2* and *Csf3* in BBB endothelial cells. Following 16h of IL-1β stimulation, BBB endothelial cells displayed a significantly increased *Csf2* (one way ANOVA – F (2,6) = 8.97, p = 0.02) and *Csf3* relative mRNA expression (one way ANOVA – F (2, 6) = 5.203, p = 0.05) compared to unstimulated and TNF-α+IFN-γ BBB endothelial cells. The relative mRNA expression of *Nos2* and *Arg1* was calculated based on the 2^-ΔΔCt^ method using *S16* **(A, B)** or *Hprt* **(C)** as reference genes. Data is presented as fold increase relative to M^unpolarized^ condition **(A, B)** or to unstimulated condition **(C)**; displayed are means and SEM from three individual experiments, *p < 0.05, **p < 0.01, ***p < 0.001, ****p < 0.0001. A mean of the raw cycle threshold (Ct) values from the independent experiments performed are provided in **Supplementary Table 3** and **4**. **(D)** mRNA expression of *Arg1*, *Tpi1* and *Gpi1* genes in MoCs following 7h incubation with BBB endothelial cells or ChP epithelial cells in the presence or absence of G-CSF and GM-CSF neutralizing antibodies. G-CSF + GM-CSF neutralizing antibodies (10µg/ml each) or isotype control (20µg/ml) were supplemented to MoCs incubated with BBB endothelial cells or ChP epithelial cells. A non-significant decrease in *Arg1*, *Tpi1 and Gpi1* mRNA expression was observed in MoCs following blocking of G-CSF and GM-CSF in IL-1β BBB endothelial cells (Arg1: IL-1β^Isotype^ Mean = 7.80, SEM = 3.29; IL-1β^G-CSF+GM-CSF^ Mean = 5.34, SEM = 1.45; Tpi1: IL-1β^Isotype^ Mean = 1.15, SEM = 0.02; IL-1β^G-CSF+GM-CSF^ Mean = 0.97, SEM = 0.12; Gpi1: IL-1β^Isotype^ Mean = 1.21, SEM = 0.11; IL-1β^G-CSF+GM-CSF^ Mean = 0.69, SEM = 0.23). Target genes were normalized to the reference gene *S16* and the data is presented as fold increase relative to the unstimulated condition (2^-ΔΔCt^). Displayed are means ± SEM from technical triplicates, 4 independent experiments. Raw cycle threshold (Ct) values from the independent experiments performed are provided in **Supplementary Table 5**. **(E)** *Arg1* relative mRNA expression in MoCs incubated for 7h with recombinant mouse G-CSF or GM-CSF proteins at different concentrations (10ng/ml, 50ng/ml or 100ng/ml). An increase in *Arg1* expression was observed upon MoC incubation with GM-CSF (MoCs + 10ng Mean = 7.48, SEM = 4.36; MoCs + 50ng Mean = 9.0, SEM = 4.95; MoCs + 100ng Mean = 7.42, SEM = 3.71). Target genes were normalized to the reference gene *Hprt* and data presented as fold increase relative to the unstimulated condition (2^-ΔΔCt^). Displayed are means ± SEM from technical triplicates, 3 independent experiments. Raw cycle threshold (Ct) values from the independent experiments performed are provided in **Supplementary Table 6**.

To understand whether these changes paralleled the acquisition of a pro- or anti-inflammatory phenotype, we analyzed expression of *Nos2* and *Arg1* in MoCs and observed strongly increased (albeit not significantly) *Nos2* expression in M^unpolarized^ cells incubated with TNF-α+IFN-γ activated BBB endothelial cells (**Figure 4A** and **Supplementary Table 3**). *Nos2* was instead significantly upregulated in M^unpolarized^ cells incubated with IFN-γ stimulated ChP epithelial cells (**Figure 4B** and **Supplementary Table 3**). Conversely, IL-1β stimulated BBB endothelial cells led to a highly significant upregulation of *Arg1* (**Figure 4A** and **Supplementary Table 3**). Increased *Arg1* expression was also observed in MoCs incubated with ChP epithelial cells in unstimulated and TNF-α stimulated conditions, albeit at a lower level (**Figure 4B** and **Supplementary Table 3**).

In conclusion, our data indicates that CNS barrier cells regulate the balance between *Nos2* and *Arg1* expression and thus trigger the acquisition of distinct pro- and anti-inflammatory functional states in interacting MoCs in a stimulus-dependent manner.

### Endothelial Derived GM-CSF Aids *Arg1* Expression in BBB-Interacting MoCs

To define the molecular mechanism behind anti-inflammatory polarization of MoCs at CNS barriers, we investigated factors secreted by *Arg1*-inducing IL1β-stimulated BBB endothelial cells (**Figure 4A**) and observed in these cells selective upregulation of *Csf3*, encoding for granulocyte colony-stimulating factor (G-CSF) and *Csf2*, encoding for granulocyte-macrophage colony-stimulating factor (GM-CSF) (**Figure 4C** and **Supplementary Table 4**). Activated endothelial cells release high amounts of G-CSF (42) and GM-CSF (41, 43–45), with these cytokines detected in both MS patients and EAE CNS tissue (46–48). G-CSF and GM-CSF are growth factors able to induce monocyte/macrophage proliferation (49), but their effect on MoC functions remains controversial (43, 50–52).

To understand the role of endothelial-derived G-CSF and GM-CSF on MoC functional polarization, we supplemented neutralizing antibodies against G-CSF and GM-CSF during the interaction of M^unpolarized^ cells with IL-1β stimulated BBB endothelial cells and observed a non-significant decrease of *Arg1*, *Tpi1* and *Gpi1* expression in M^unpolarized^ cells upon blocking (**Figure 4D** and **Supplementary Table 5**). In parallel, to assess the direct effect of GM-CSF and G-CSF on MoCs, we incubated these factors with M^unpolarized^ cells and observed that GM-CSF, but not G-CSF, consistently triggered (albeit not significantly) *Arg1* upregulation in M^unpolarized^ cells (**Figure 4E** and **Supplementary Table 6**).

Taken together, our *in vitro* experiments suggest that GM-CSF secreted by IL-1β stimulated endothelial cells could only in part contribute to the acquisition of an arginase-1^+^ phenotype in interacting MoCs.

### IL-1β Signaling in BBB Endothelial Cells Regulates Arginase-1 Expression in MoCs

**Figure 5:**
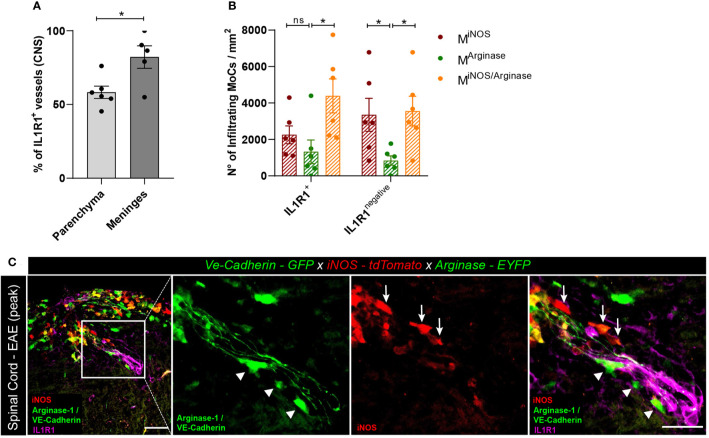
Polarized MoC density around IL1R1^+^ and IL1R1^negative^ vasculature during EAE. **(A)** Relative percentage of IL1R1^+^ and IL1R1^negative^ vessels in the inflamed spinal cord parenchyma (n=6) and leptomeninges (n=5) of *VE-cadherin-GFP x iNOS-tdTomato x Arginase-EYFP* mice induced with EAE (3 days after disease onset). A higher number of IL1R1^+^ vessels were detected in the meninges compared to parenchyma, unpaired t test (p=0.018, *p < 0.05). The total amount of vessels in tissue sections was assessed using the GFP positivity of VE-Cadherin expressing endothelial cells. Immunostaining with IL1R1-specific antibody revealed receptor expression. **(B)** Density of M^iNOS^, M^Arginase^ and M^iNOS/Arginase^ cells surrounding IL1R1^+^ and IL1R1^negative^ vasculature of *VE-cadherin-GFP x iNOS-tdTomato x Arginase-EYFP* mice induced with EAE (6 mice, 3 days after disease onset). The quantified areas include tissue at a maximum distance of 20 µm from the endothelial GFP signal. Data represented as mean ± SEM. In the proximity of IL1R1^+^ vessels, a higher density of M^iNOS/Arginase^ cells (Mean = 4389, SEM = 933.6) are detected as compared to M^Arginase^ (Mean = 1321, SEM = 650.9) (p = 0.136) and to M^iNOS^ (Mean = 2254, SEM = 490.6) (p = 0.105). In contrast, in the proximity of IL1R1^negative^ vessels, a higher number of M^iNOS^ (Mean = 3352, SEM = 906.1) (p=0.048) and M^iNOS/Arginase^ cells (Mean = 3556, SEM = 812.3) (p=0.031) are detected as compared to M^Arginase^ cells (Mean = 839.6, SEM = 258.0). Two-way ANOVA with Tukey´s multiple comparisons test, *p < 0.05, ns, not significant. **(C)** Representative confocal images of spinal cord tissue from *VE-cadherin-GFP x iNOS-tdTomato x Arginase-EYFP* mice induced with EAE (3 days after disease onset). tdTomato indicates M^iNOS^, EYFP M^Arginase^ cells and GFP VE-cadherin expression at BBB endothelial cell junctions. Immunostaining with IL1R1-specific antibody reveals IL1R1 receptor expression. Arrowheads highlight M^Arginase^ cells and arrows indicate M^iNOS^ cells in the perivascular space and in the tissue surrounding the vessel. Scale bar, 50 μm; magnified regions, scale bar 30 μm.

The induction of pro- and anti-inflammatory genes in MoCs interacting with CNS barrier cells suggests that the migration of MoCs across CNS borders can directly regulate the phenotype of invading MoCs. To understand whether IL-1β signaling in BBB cells triggers the expression of arginase-1 in MoCs as observed *in vitro*, we induced EAE in *VE-cadherin-GFP x iNOS-tdTomato x Arginase-EYFP* mice and immune-stained spinal cord sections with IL-1β receptor (IL1R1)-specific antibodies. As suggested by a previous report (41), we observed diffuse IL1R1 immunostaining in the leptomeningeal vasculature, whereas a lower proportion of IL1R1^+^ vasculature could be detected in the inflamed spinal cord parenchyma (**Figure 5A**). Secondly, we compared the density and distribution of M^iNOS^, M^Arginase^ and M^iNOS/Arginase^ cells lining IL1R1^+^ or IL1R1^negative^ vasculature within spinal cord lesions. Given their position, we assumed that analyzed MoCs recently reached the CNS following interaction with proximal endothelial cells. Interestingly, while IL1R1^negative^ vasculature showed a statistically higher presence of M^iNOS^ compared to M^Arginase^ cells, IL1R1^+^ vasculature was surrounded by an equal number of M^iNOS^ and M^Arginase^ cells. Compared to M^Arginase^ cells however, transitional M^iNOS/Arginase^ cells accumulated more densely around all vessels (**Figures 5B, C**).

Taken together, both our *in vitro* and *in vivo* observations support a role of IL-1β signaling in endothelial cells in the regulation of MoC functional specifications during CNS invasion.

## Discussion

CNS inflammation as observed in MS and in EAE drives the mobilization of monocytes from the bone marrow to the bloodstream (53, 54), with cells accessing the CNS at the level of the BBB, the blood-CSF barrier, or at the subarachnoid vasculature within the leptomeninges (1). Accumulation of MoCs at CNS borders is thus a key event in disease development (23, 55). While the study of immune cell migration through distinct CNS access gateways has considerably increased the efficacy of MS treatments (56), research has however largely focused on lymphocyte dynamics (57–59), with the trafficking routes of circulating monocytes during neuroinflammation remaining surprisingly unclear (24). Furthermore, the anatomical sites and the mechanisms leading to the acquisition of pro- or anti-inflammatory specifications in CNS-invading MoCs have not been properly investigated.

In this study, we used bone marrow-derived M^unpolarized^, M^LPS+IFN-y^ and M^IL-4+IL-13^ cells to model the distinct *in vivo* functional features of MoCs and to assess their interaction and transmigration properties with *in vitro* BBB endothelial and blood-CSF barrier epithelial cells.

Activation of endothelial cells augmented adhesion and diapedesis of M^unpolarized^ cells, suggesting that inflammation increases the extravasation of MoCs as shown for other immune cells (21, 60, 61). While inflammatory conditions decreased endothelial TEER implying impaired barrier properties, junctional continuity remained largely intact, in line with the notion that BBB physical disruption is not strictly needed for cell extravasation (62).

Notably, polarization of MoCs toward pro-inflammatory M^LPS+IFN-y^ or anti-inflammatory M^IL-4+IL-13^ states drastically reduced cellular adhesion to endothelial cells, in line with previous data showing that functionally-committed MoCs are not present in peripheral blood and lymph nodes during EAE (17). The observed downregulation of *Ccr2* might contribute to the reduced adhesion and migration of M^LPS+IFN-y^ cells (63, 64). Interestingly, a similar downregulation of *Ccr2* in iNOS^+^ pro-inflammatory CNS-invading macrophages was observed during EAE (data not shown) (17). Nonetheless, despite similar chemokine receptor levels and increased β2 integrin expression compared to M^unpolarized^ cells, also M^IL-4+IL-13^ cells failed to efficiently interact with BBB endothelial cells. Other adhesion factors, signaling molecules and physical characteristics can thus affect the dynamics of MoCs at the BBB, but the overall mechanism remains unclear.

While the acquisition of a full functional specification is not a pre-requisite for MoC extravasation at the BBB, subsequent accumulation in the perivascular space seems associated with a preferential pro-inflammatory glycolytic state in MoCs, as shown in both EAE and MS (17, 65). Crossing of activated endothelial cells towards the perivascular space milieu might therefore represent a key step in the functional priming of MoCs before invasion of the CNS parenchyma. By analyzing macrophage distribution in the *VE-cadherin-GFP x iNOS-tdTomato x Arginase-EYFP* model upon EAE induction, we could accordingly observe an increased accumulation of pro-inflammatory M^iNOS^ cells in spinal cord perivascular spaces compared to other CNS interfaces such as leptomeninges and ChP stroma. Notably, in a different series of *in vitro* experiments, *Nos2* expression could be specifically induced (albeit not significantly) in M^unpolarized^ cells by IFN-γ-stimulated BBB endothelial cells. This observation suggests that, in an IFN-γ (T cell-) dominated inflammatory context, passage through the BBB can aid priming of MoCs toward a pro-inflammatory state.

Once accumulating in the CNS however, M^iNOS^ cells evolve their phenotype by upregulating *Arg1* expression and becoming M^iNOS/Arginase^ cells (17). Furthermore, distinct M^Arginase^ cells invade the CNS parenchyma without previously expressing *Nos2* (17). Several factors might be responsible for driving *Arg1* expression and the phenotypic change in M^iNOS^ cells, including astrocyte-secreted molecules (17). Our *in vitro* work now showed that IL-1β signaling in BBB endothelial cells is a key driver of *Arg1* expression in CNS-infiltrating MoCs. Accordingly, analysis of parenchymal lesions in the EAE model indicated that MoCs accumulate in different proportions nearby IL1R1^+^ vessels compared to IL1R1^negative^ vessels. At the same time, a slightly increased presence of “phenotype-shifting” M^iNOS/Arginase^ cells compared to perivascular spaces could be observed in the leptomeninges, a compartment characterized by higher density of IL1R1^+^ vasculature.

Taken together, depending on the anatomical location and on the activation of the BBB endothelial cells encountered during migration, MoCs can be primed toward an iNOS^+^ pro-inflammatory or arginase-1^+^ anti-inflammatory state while accumulating at this CNS barrier. Moreover, our *in vitro* model suggests that the production of GM-CSF by an IL-1β activated endothelium might contribute to the *Arg1* expression observed in MoCs, however not exclusively. GM-CSF release by IL-1β activated CNS endothelial cells was previously reported to enhance CCR2^+^ MoC activation (43). Upregulation of arginase-1 by recombinant GM-CSF in MoCs has also been described (66), but the connection between these signaling axes during neuroinflammation was not established. While the GM-CSF-mediated upregulation of the anti-inflammatory gene *Arg1* appears somewhat in contrast with the disease-driving function of GM-CSF in neuroinflammatory models (67, 68), MoCs stimulated with GM-CSF have been described as cells sharing pro- and anti-inflammatory characteristics (50–52), with a recent work even proposing that exposure to GM-CSF can lead to the formation of monocyte-derived suppressor cells (69).

Functional polarization could also be triggered by the interaction of MoCs with primary ChP epithelial cells. Similar to what observed with the BBB model, IFN-γ stimulation allowed ChP epithelial cells to drive *Nos2* expression in MoCs, while *Arg1* was induced following MoC interaction with unstimulated and TNF-α stimulated epithelial cells, and less strongly by IFN-γ stimulated epithelial cells. In contrast with what we observed at the BBB however, M^unpolarized^ and polarized M^IL-4+IL-13^ and M^LPS+IFN-y^ macrophages could efficiently adhere to and move across ChP epithelial cells. Taken together, these experiments suggested that the blood-CSF barrier could constitute a CNS access gateway and a priming site for both pro- and anti-inflammatory MoCs. Nonetheless, when analyzing brain sections from mice induced with EAE, only low numbers of iNOS^+^/arginase-1^+^ MoCs could be observed *in situ*, compared to the high density of CCR2^+^ MoCs detected within the ChP. Thus, local acquisition of an overt pro- or anti-inflammatory phenotype can happen within the ChP stroma, but remains a minor phenomenon compared to the recruitment of yet-to-be-polarized MoCs. Intravital studies are still crucially needed to shed light on the real MoC dynamics at the secluded ChP.

In conclusion, our work sheds light on the dynamics of MoC recruitment at the different borders of the CNS during neuroinflammation. Collectively, our data indicate that local signaling cues and interaction between MoCs and CNS barrier cells can significantly shape the function of invading cell and thus affect the pathological and clinical evolution of autoimmune CNS inflammation.

## Data Availability Statement

The original contributions presented in the study are included in the article/**Supplementary Material**. Further inquiries can be directed to the corresponding author.

## Ethics Statement

The animal study was reviewed and approved by Veterinary office of the Canton of Bern, Switzerland.

## Author Contributions

GL and DI designed the experiments. DI and SW performed and analyzed all experiments, GL and DI co-wrote the manuscript. GL supervised the study. All authors contributed to the article and approved the submitted version.

## Funding

This work is supported by a Swiss Multiple Sclerosis Society grant, Italian MS grant (FISM 2019/R-Single/001) and by Scherbarth Foundation funding awarded to GL.

## Conflict of Interest

The authors declare that the research was conducted in the absence of any commercial or financial relationships that could be construed as a potential conflict of interest.

## Supplementary Material

Supplementary Figure 1 Primary Mouse Brain Microvascular Endothelial Cells characterisation. BBB endothelial cells were isolated from C57BL/6J male mice and stimulated with 20ng/ml IL-1β or with 5ng/ml TNF-a +100IU/ml IFN-γ. Unstimulated BBB endothelial cells were used as control. **(A–D)** Immunofluorescence stainings of adherens junction molecules β-catenin **(A)** and VE-cadherin **(B)** and of the tight junction molecules ZO-1 **(C)** and claudin-5 **(D)**, scale bar 50μm. Pictures are representative of three independent experiments. **(E–J)** Immunofluorescence stainings of ICAM-1 **(E)**, VCAM-1 **(F)** and E-selectin **(G)** in unstimulated, IL-1β and TNF-α+IFN-γ stimulated BBB endothelial cells at culture day seven. Pictures are representative of three independent experiments, 50μm scale bar. **(H–J)** Quantification of mean fluorescence intensity of ICAM-1, VCAM-1 and E-selectin immunofluorescence stainings of the BBB endothelial monolayer performed using ImageJ software. **(H)** ICAM-1 expression is significantly upregulated in IL-1β BBB endothelial cells compared to both unstimulated (p=0.005) and TNF-α+IFN-γ BBB endothelial cells (one-way ANOVA p=0.007) (n=2). **(I)** VCAM-1 mean fluorescence intensity is higher on both IL-1β (Mean = 27.82, SEM = 2.58) and TNF-α+IFN-γ (Mean = 26.15, SEM = 3) BBB endothelial cells (one-way ANOVA F (2,6) = 2.504, p= 0.162) (n=3). **(J)** E-selectin mean fluorescence intensity is significantly upregulated in IL-1β BBB endothelial cells compared to both unstimulated (one-way ANOVA, p=0.004) and TNF-α+IFN-γ BBB endothelial cells (one-way ANOVA, p=0.005) (n=3). **(K)** Tightness of BBB endothelial cells cultured on 5μm pore size filters was determined by TEER (Ω*cm^2^). Data is presented as mean and standard error of the mean (SEM) from five independent experiments. TEER of stimulated BBB endothelial cells is significantly lower compared to unstimulated endothelium (one-way ANOVA, F (2,42) = 35.86, *p* < 0.0001).

Supplementary Figure 2 Characterization of Primary Mouse Choroid Plexus Epithelial Cells. ChP epithelial cells were isolated from lateral, 3^rd^ and 4^th^ brain ventricles of C57BL/6J male mice and at culture day 6 were stimulated with 10ng/ml TNF-α or with 100IU/ml IFN-γ for 16h. Unstimulated ChP epithelial cells were used as control condition. Shown are immunofluorescence stainings of adherens junction molecules E-cadherin **(A)**, β-catenin **(B)** and tight junction molecule junctional adhesion molecule-A (JAM-A) **(C)** expressed on unstimulated and TNF-α or IFN-γ stimulated ChP epithelial cells at culture day seven. Pictures are representative of two independent experiments for ICAM-1 and one experiment for VCAM-1, scale bar 50μm. **(D, E)** Surface expression of ICAM-1 and VCAM-1 in unstimulated and TNF-α or IFN-γ stimulated ChP epithelial cells at culture day seven, representative images from 2 experiments (ICAM-1) and one experiment (VCAM-1). **(F, G)** Quantification of the mean fluorescence intensity of ICAM-1 and VCAM-1 immunofluorescence stainings of ChP epithelial monolayer performed using ImageJ software. Both ICAM-1 (n=2) and VCAM-1 (n=1) are upregulated in IFN-γ ChP epithelial cells compared to unstimulated and TNF-α conditions (ICAM-1 Unstimulated vs IFN-γ stimulated ChP epithelial cells p = 0.035, one way ANOVA). **(D)** Tightness of ChP epithelial cells cultured on 5μm pore size filters was determined by TEER (Ω*cm^2^). No statistically significant differences were detected between conditions, one-way ANOVA, four independent experiments.

Supplementary Figure 3 Bone marrow-derived macrophages were isolated from C57BL/6J male mice and stimulated with 100ng/ml LPS + 10ng/ml IFN-γ (M^LPS+IFN-γ^) or with 10ng/ml IL-4 + 10ng IL-13 (M^IL-4+IL-13^). M^unpolarized^ cells were left unstimulated. **(A–C)** Gene expression was assessed by RTqPCR following mRNA extraction and cDNA synthesis. Data was normalized using *Hprt* as reference gene and presented as fold increase relative to M^unpolarized^ condition (2^-ΔΔCt^, see Methods). Raw cycle threshold (Ct) values from the independent experiments performed are provided in **Supplementary Table 2**. **(A)** mRNA expression of pro-inflammatory genes *Nos2, Fpr2, Gpr18, Cd38, Cd86, Stat1* and of **(B)** anti-inflammatory genes *Retnla, Arg1, Mrc1, Ym1* and *Egr2* is shown. Displayed are means ± SEM obtained from technical triplicates, 3 independent experiments; one-way ANOVA analysis with Tukey´s multiple comparisons test. M^LPS+IFN-γ^ compared to M^IL-4+Il-13^ and to M^unpolarized^ cells have a significantly higher expression of *Nos2* (F (2,18) = 4.677, *p* = 0.023)*, Fpr2* (F (2,6) = 6.587, *p* = 0.031)*, Cd38* (F (2,6) = 46.46, *p* =0.0002)*, Cd86* (F (2,6) = 114.5, *p* < 0.0001)*, Stat1* (F (2,6) = 63.48, *p* < 0.0001), *Gpr18* (F (2,6) = 4.012, *p* < 0.078). Compared to M^unpolarized^ and M^LPS+IFN-γ^ macrophages, M^IL-4+IL-13^ cells display significantly higher expression of *Retnla* (F (2,3) = 1.163, *p* = 0.423), *Arg1* [F (2,18) = 22.13, *p* < 0.0001], *Ym1* (F (2,6) = 3.120, *p* = 0.117) *Egr2* (F (2,6) = 12.68, *p* = 0.007) and *Mrc1* (one-way ANOVA, F (2,6) = 30.89, *p* = 0.007). **(C)** Relative expression of *Ccr1*, *Ccr2* and *Ccr5* in M^unpolarized^, M^LPS+IFN-γ^, M^IL-4+Il-13^ cells after 48h cytokine stimulation. A statistically significant increase in *Ccr1* expression was observed in M^LPS+IFN-γ^ compared to M^unpolarized^ cells (Tukey´s test: p = 0.03). Conversely, M^LPS+IFN-γ^ displayed significantly downregulated *Ccr2* expression compared to M^unpolarized^ cells (Tukey´s test: p = 0.001) and to M^IL-4+IL-13^ cells (Tukey´s test: p = 0.01). Displayed are mean ± SEM from 3 independent experiments. **(D)** Surface expression of β2, α4 and β1 integrin molecules detected on M^unpolarized^, M^LPS+IFN-γ^ and M^IL-4+Il-13^ macrophages by flow cytometry. M^IL-4+Il-13^ display a significantly increased β2 integrin expression compared to M^LPS+IFN-γ^ and M^unpolarized^ cells (one-way ANOVA, F (2,3) = 14.92, *p* = 0.0276). Displayed are relative mean fluorescence intensity (MFI) values obtained by subtracting the MFI of stained samples from the MFI of isotype control-stained samples; data is presented as mean ± SEM from 2 independent experiments for β2 integrin and from 4 independent experiments for α4 and β1 integrins.

Supplementary Figure 4 The influence of BBB endothelial and ChP epithelial cells on MoC activation. MoCs were incubated with BBB endothelial cells and ChP epithelial cells for 7h and afterwards, they were recovered from the monolayers and their mRNA was isolated. Shown are relative mRNA expressions of *Tpi1*, *Gpi1* **(A, B)**, *Aldoa*, *Pfkl* **(C, D)**, *Il6*, *Il6r* **(E, F)**, *Mmp2 and Il1β* **(G, H)** genes. Data was normalized using *Hprt* as reference gene and is presented as fold increase relative to M^unpolarized^ condition (2^-ΔΔCt^, see Methods). Displayed are means ± SEM obtained from technical triplicates, 3 independent experiments, one-way ANOVA analysis with Tukey´s multiple comparison. **(A, C, E, G)** MoCs incubated with cytokine activated BBB endothelial cells showed an increased expression in the following genes as compared to M^unpolarized^ cells: **(A)** *Tpi1 -* F (3, 8) = 9.492, *p* = 0.0052 (Tukey´s test: M^unpolarized^ vs M^IL-1β Endothelium^ p = 0.004; M^unpolarized^ vs M^TNF-α+IFN-γ Endothelium^ p = 0.0178); *Gpi1 -* F (3, 8) = 11.45, *p* = 0.0029 (Tukey´s test: M^unpolarized^ vs M^Unstimulated Endothelium^ p = 0.0449; M^unpolarized^ vs M^IL-1β Endothelium^ p = 0.0018; M^unpolarized^ vs M^TNF-α+IFN-γ Endothelium^ p = 0.029); **(C)** *Aldoa -* F (3, 8) = 6.105, *p* = 0.018 (Tukey´s test: M^unpolarized^ vs M^IL-1β Endothelium^ p = 0.0225); **(E)** *Il6r -* F (3, 8) = 4.467, *p* = 0.04 (Tukey´s test: M^unpolarized^ vs M^IL-1β Endothelium^ p = 0.047); **(G)** *Il1β -* F (3, 8) = 7.009, *p* = 0.0125 (Tukey´s test: M^unpolarized^ vs M^IL-1β Endothelium^ p = 0.0132). Additionally, statistically significant differences were found in *Il1β* (p = 0.028) and *Aldoa* (p = 0.038) gene expressions between M^unstimulated Endothelium^ vs M^IL-1β Endothelium^. *Pfkl, Il6* and *Mmp* were also increased in MoCs upon incubation with BBB endothelial cells, however statistical significance was not reached. **(B, D, F, H)** Following MoC incubation with ChP epithelial cells, we observed the following changes in gene expression: compared to M^unpolarized^ cells, MoCs incubated with cytokine activated ChP epithelial cells showed an increased mRNA expression in **(B)** *Tpi1 -* F (3, 8) = 10.36, *p* = 0.004 (Tukey´s test: M^unpolarized^ vs M^unstimulated Epithelium^ p = 0.0045; M^unpolarized^ vs M^TNF-α Epithelium^ p = 0.0292; M^unpolarized^ vs M^IFN-γ Epithelium^ p = 0.008); *Gpi1 -* F (3, 8) = 10.46, *p* = 0.0038 (Tukey´s test: M^unpolarized^ vs M^unstimulated Epithelium^ p = 0.0049; M^unpolarized^ vs M^TNF-α Epithelium^ p = 0.0063; M^unpolarized^ vs M^IFN-γ Epithelium^ p = 0.0458); **(D)** *Pfkl -* F (3, 8) = 5.186, *p* = 0.028 (Tukey´s test: M^unpolarized^ vs M^unstimulated Epithelium^ p = 0.032; M^unpolarized^ vs M^TNF-α Epithelium^ p = 0.046). **(D)** *Aldoa*, **(F)** *Il6r* and **(H)** *Mmp2* were also increased in MoCs upon incubation with ChP epithelial cells, however statistical significance was not reached. Raw cycle threshold (Ct) values from the independent experiments performed are provided in **Supplementary Table 3**.

Supplementary Tables 1–6 Fiji Macro used for the automatic quantification of macrophages attached on the luminal side of BBB endothelial cells or on the upper side of ChP epithelial cells filters *in vitro.* Fiji Macro used for the automatic quantification of CX3CR1^+^ and CCR2^+^ cells in the choroid plexus of *CX3CR1-GFP x CCR2-RFP* mice.
