# Supplementary figures and images for: Central Nervous System Barriers Impact Distribution and Expression of iNOS and Arginase-1 in Infiltrating Macrophages During Neuroinflammation
Source: Front Immunol. 2021 Apr 15;12:666961. doi: 10.3389/fimmu.2021.666961 (PMC8082146; doi:10.3389/fimmu.2021.666961)

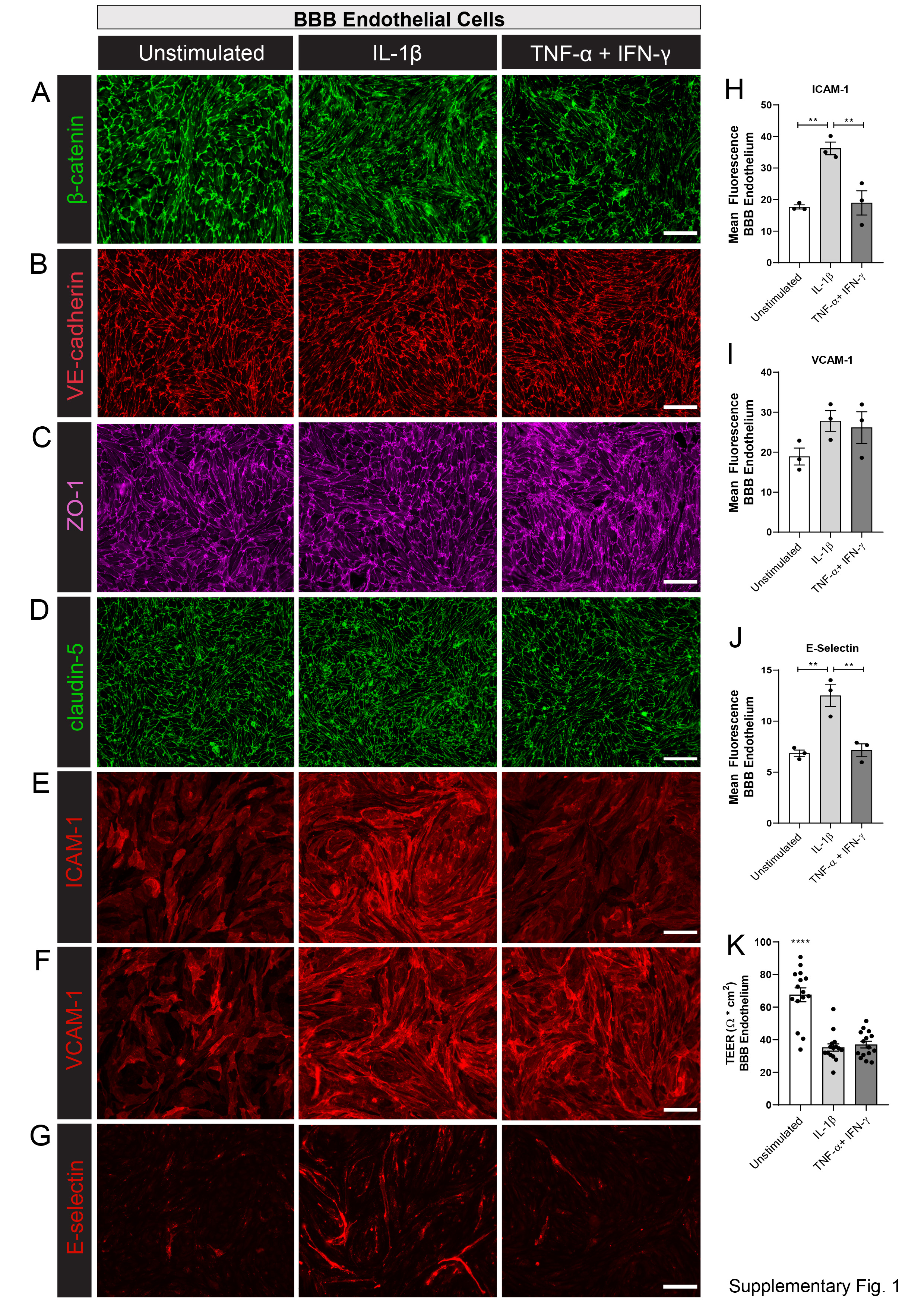

Supplement: Supplementary Figure 1 — Primary Mouse Brain Microvascular Endothelial Cells characterisation. BBB endothelial cells were isolated from C57BL/6J male mice and stimulated with 20ng/ml IL-1β or with 5ng/ml TNF-a +100IU/ml IFN-γ. Unstimulated BBB endothelial cells were used as control. (A–D) Immunofluorescence stainings of adherens junction molecules β-catenin (A) and VE-cadherin (B) and of the tight junction molecules ZO-1 (C) and claudin-5 (D), scale bar 50μm. Pictures are representative of three independent experiments. (E–J) Immunofluorescence stainings of ICAM-1 (E), VCAM-1 (F) and E-selectin (G) in unstimulated, IL-1β and TNF-α+IFN-γ stimulated BBB endothelial cells at culture day seven. Pictures are representative of three independent experiments, 50μm scale bar. (H–J) Quantification of mean fluorescence intensity of ICAM-1, VCAM-1 and E-selectin immunofluorescence stainings of the BBB endothelial monolayer performed using ImageJ software. (H) ICAM-1 expression is significantly upregulated in IL-1β BBB endothelial cells compared to both unstimulated (p=0.005) and TNF-α+IFN-γ BBB endothelial cells (one-way ANOVA p=0.007) (n=2). (I) VCAM-1 mean fluorescence intensity is higher on both IL-1β (Mean = 27.82, SEM = 2.58) and TNF-α+IFN-γ (Mean = 26.15, SEM = 3) BBB endothelial cells (one-way ANOVA F (2,6) = 2.504, p= 0.162) (n=3). (J) E-selectin mean fluorescence intensity is significantly upregulated in IL-1β BBB endothelial cells compared to both unstimulated (one-way ANOVA, p=0.004) and TNF-α+IFN-γ BBB endothelial cells (one-way ANOVA, p=0.005) (n=3). (K) Tightness of BBB endothelial cells cultured on 5μm pore size filters was determined by TEER (Ω*cm2). Data is presented as mean and standard error of the mean (SEM) from five independent experiments. TEER of stimulated BBB endothelial cells is significantly lower compared to unstimulated endothelium (one-way ANOVA, F (2,42) = 35.86, p < 0.0001). [file Image_1.jpeg]

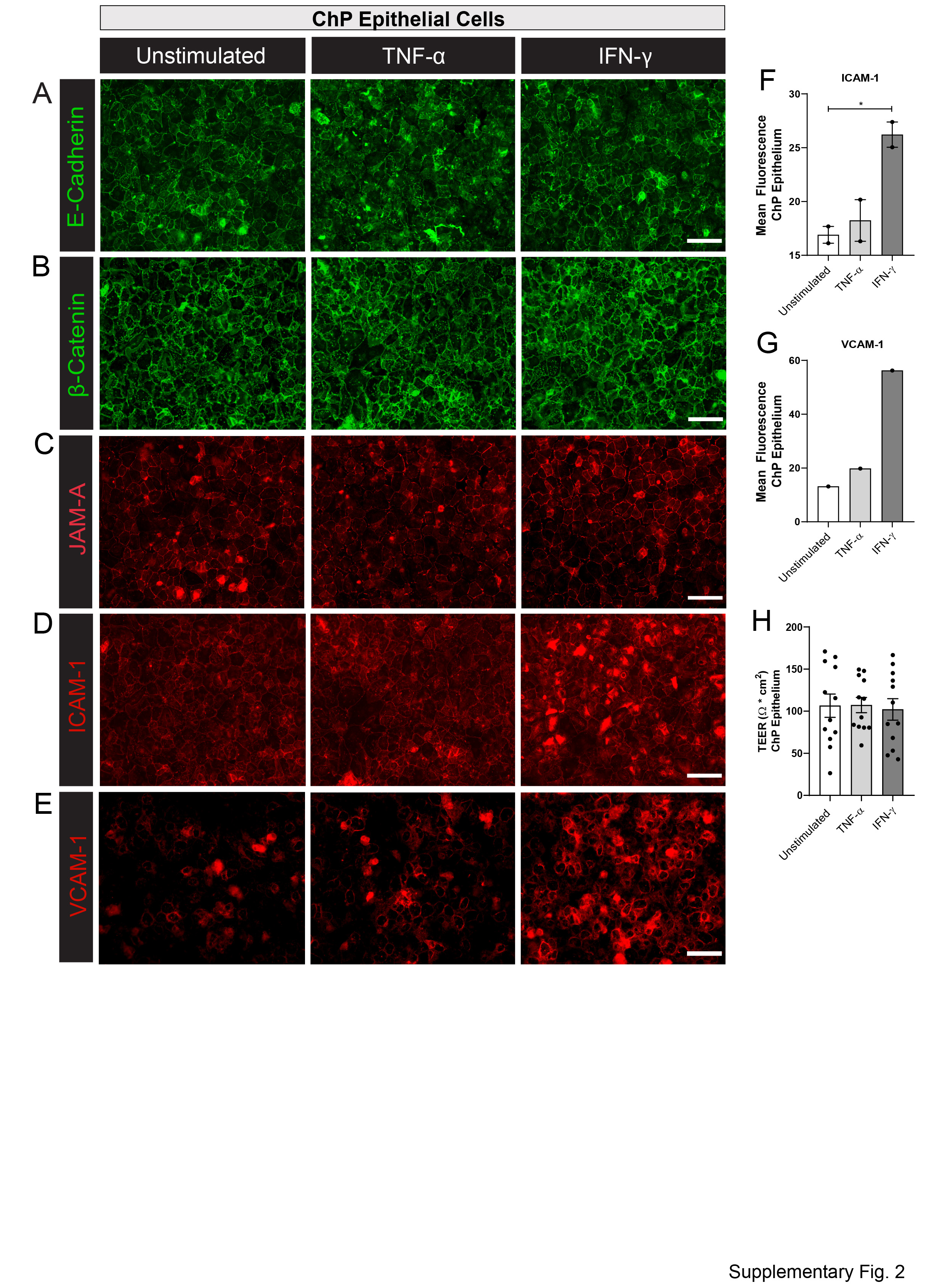

Supplement: Supplementary Figure 2 — Characterization of Primary Mouse Choroid Plexus Epithelial Cells. ChP epithelial cells were isolated from lateral, 3rd and 4th brain ventricles of C57BL/6J male mice and at culture day 6 were stimulated with 10ng/ml TNF-α or with 100IU/ml IFN-γ for 16h. Unstimulated ChP epithelial cells were used as control condition. Shown are immunofluorescence stainings of adherens junction molecules E-cadherin (A), β-catenin (B) and tight junction molecule junctional adhesion molecule-A (JAM-A) (C) expressed on unstimulated and TNF-α or IFN-γ stimulated ChP epithelial cells at culture day seven. Pictures are representative of two independent experiments for ICAM-1 and one experiment for VCAM-1, scale bar 50μm. (D, E) Surface expression of ICAM-1 and VCAM-1 in unstimulated and TNF-α or IFN-γ stimulated ChP epithelial cells at culture day seven, representative images from 2 experiments (ICAM-1) and one experiment (VCAM-1). (F, G) Quantification of the mean fluorescence intensity of ICAM-1 and VCAM-1 immunofluorescence stainings of ChP epithelial monolayer performed using ImageJ software. Both ICAM-1 (n=2) and VCAM-1 (n=1) are upregulated in IFN-γ ChP epithelial cells compared to unstimulated and TNF-α conditions (ICAM-1 Unstimulated vs IFN-γ stimulated ChP epithelial cells p = 0.035, one way ANOVA). (D) Tightness of ChP epithelial cells cultured on 5μm pore size filters was determined by TEER (Ω*cm2). No statistically significant differences were detected between conditions, one-way ANOVA, four independent experiments. [file Image_2.jpeg]

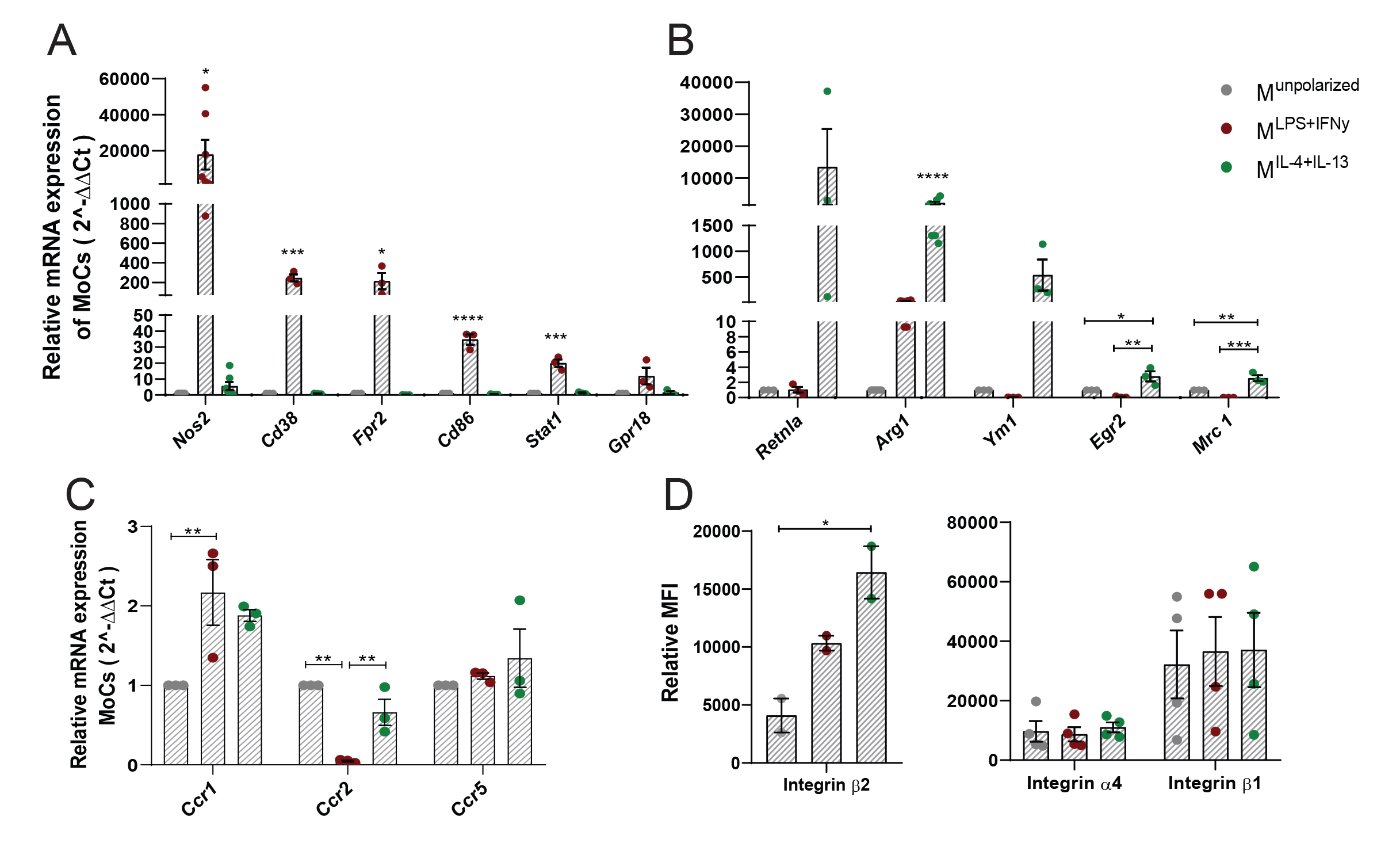

Supplement: Supplementary Figure 3 — Bone marrow-derived macrophages were isolated from C57BL/6J male mice and stimulated with 100ng/ml LPS + 10ng/ml IFN-γ (MLPS+IFN-γ) or with 10ng/ml IL-4 + 10ng IL-13 (MIL-4+IL-13). Munpolarized cells were left unstimulated. (A–C) Gene expression was assessed by RTqPCR following mRNA extraction and cDNA synthesis. Data was normalized using Hprt as reference gene and presented as fold increase relative to Munpolarized condition (2-ΔΔCt, see Methods). Raw cycle threshold (Ct) values from the independent experiments performed are provided in Supplementary Table 2 . (A) mRNA expression of pro-inflammatory genes Nos2, Fpr2, Gpr18, Cd38, Cd86, Stat1 and of (B) anti-inflammatory genes Retnla, Arg1, Mrc1, Ym1 and Egr2 is shown. Displayed are means ± SEM obtained from technical triplicates, 3 independent experiments; one-way ANOVA analysis with Tukey´s multiple comparisons test. MLPS+IFN-γ compared to MIL-4+Il-13 and to Munpolarized cells have a significantly higher expression of Nos2 (F (2,18) = 4.677, p = 0.023), Fpr2 (F (2,6) = 6.587, p = 0.031), Cd38 (F (2,6) = 46.46, p =0.0002), Cd86 (F (2,6) = 114.5, p < 0.0001), Stat1 (F (2,6) = 63.48, p < 0.0001), Gpr18 (F (2,6) = 4.012, p < 0.078). Compared to Munpolarized and MLPS+IFN-γ macrophages, MIL-4+IL-13 cells display significantly higher expression of Retnla (F (2,3) = 1.163, p = 0.423), Arg1 [F (2,18) = 22.13, p < 0.0001], Ym1 (F (2,6) = 3.120, p = 0.117) Egr2 (F (2,6) = 12.68, p = 0.007) and Mrc1 (one-way ANOVA, F (2,6) = 30.89, p = 0.007). (C) Relative expression of Ccr1, Ccr2 and Ccr5 in Munpolarized, MLPS+IFN-γ, MIL-4+Il-13 cells after 48h cytokine stimulation. A statistically significant increase in Ccr1 expression was observed in MLPS+IFN-γ compared to Munpolarized cells (Tukey´s test: p = 0.03). Conversely, MLPS+IFN-γ displayed significantly downregulated Ccr2 expression compared to Munpolarized cells (Tukey´s test: p = 0.001) and to MIL-4+IL-13 cells (Tukey´s test: p = 0.01). Displayed are mean ± SEM from 3 independ [file Image_3.jpeg]

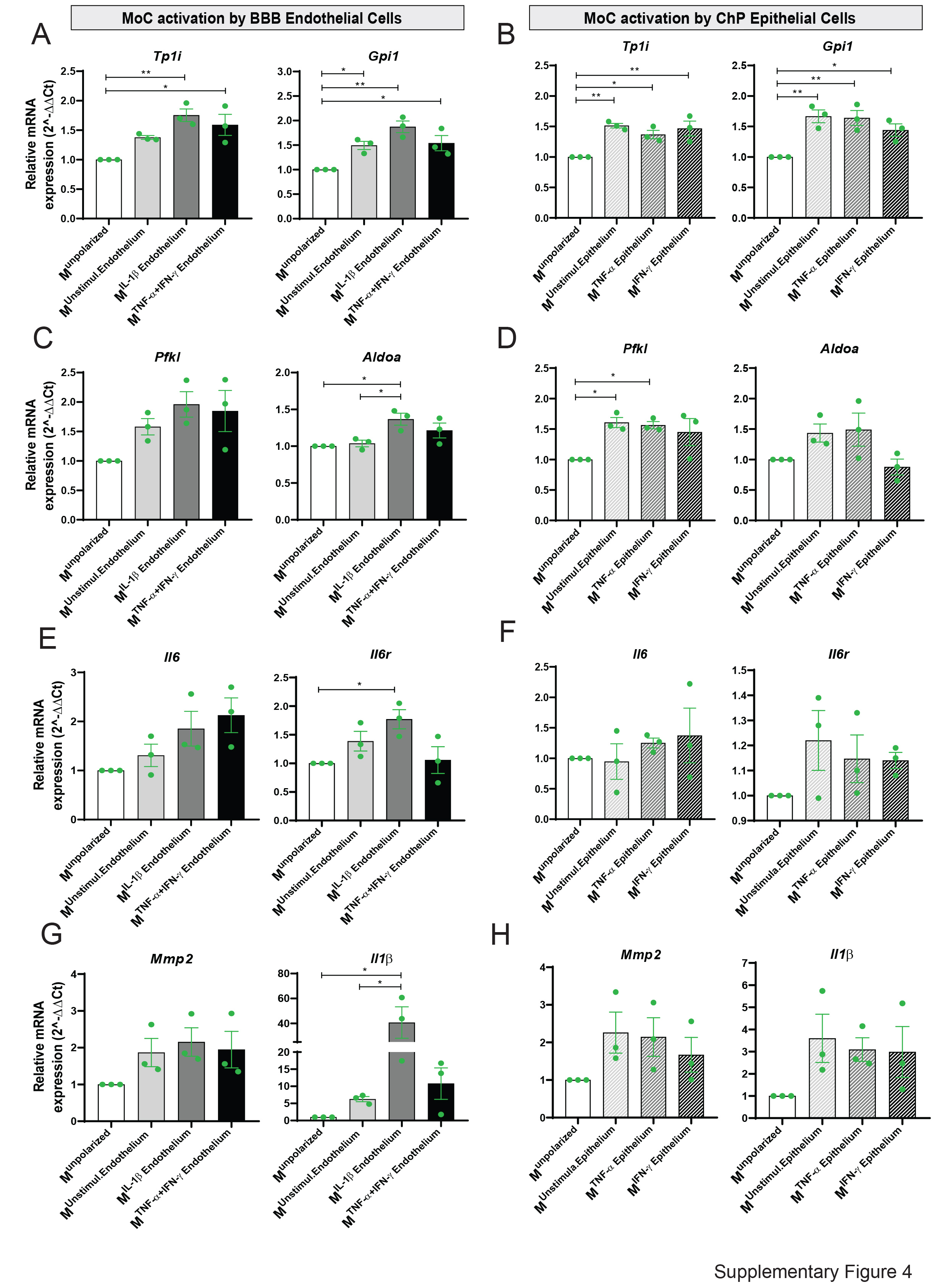

Supplement: Supplementary Figure 4 — The influence of BBB endothelial and ChP epithelial cells on MoC activation. MoCs were incubated with BBB endothelial cells and ChP epithelial cells for 7h and afterwards, they were recovered from the monolayers and their mRNA was isolated. Shown are relative mRNA expressions of Tpi1, Gpi1 (A, B), Aldoa, Pfkl (C, D), Il6, Il6r (E, F), Mmp2 and Il1β (G, H) genes. Data was normalized using Hprt as reference gene and is presented as fold increase relative to Munpolarized condition (2-ΔΔCt, see Methods). Displayed are means ± SEM obtained from technical triplicates, 3 independent experiments, one-way ANOVA analysis with Tukey´s multiple comparison. (A, C, E, G) MoCs incubated with cytokine activated BBB endothelial cells showed an increased expression in the following genes as compared to Munpolarized cells: (A) Tpi1 - F (3, 8) = 9.492, p = 0.0052 (Tukey´s test: Munpolarized vs MIL-1β Endothelium p = 0.004; Munpolarized vs MTNF-α+IFN-γ Endothelium p = 0.0178); Gpi1 - F (3, 8) = 11.45, p = 0.0029 (Tukey´s test: Munpolarized vs MUnstimulated Endothelium p = 0.0449; Munpolarized vs MIL-1β Endothelium p = 0.0018; Munpolarized vs MTNF-α+IFN-γ Endothelium p = 0.029); (C) Aldoa - F (3, 8) = 6.105, p = 0.018 (Tukey´s test: Munpolarized vs MIL-1β Endothelium p = 0.0225); (E) Il6r - F (3, 8) = 4.467, p = 0.04 (Tukey´s test: Munpolarized vs MIL-1β Endothelium p = 0.047); (G) Il1β - F (3, 8) = 7.009, p = 0.0125 (Tukey´s test: Munpolarized vs MIL-1β Endothelium p = 0.0132). Additionally, statistically significant differences were found in Il1β (p = 0.028) and Aldoa (p = 0.038) gene expressions between Munstimulated Endothelium vs MIL-1β Endothelium. Pfkl, Il6 and Mmp were also increased in MoCs upon incubation with BBB endothelial cells, however statistical significance was not reached. (B, D, F, H) Following MoC incubation with ChP epithelial cells, we observed the following changes in gene expression: compared to Munpolarized cells, MoCs incubated with cytokine activated ChP epith [file Image_4.jpeg]
