# Supplementary material for: Central Nervous System Barriers Impact Distribution and Expression of iNOS and Arginase-1 in Infiltrating Macrophages During Neuroinflammation
Source: Front Immunol. 2021 Apr 15;12:666961. doi: 10.3389/fimmu.2021.666961 (PMC8082146; doi:10.3389/fimmu.2021.666961)
Supplement: Supplementary Tables 1–6 — Fiji Macro used for the automatic quantification of macrophages attached on the luminal side of BBB endothelial cells or on the upper side of ChP epithelial cells filters in vitro. Fiji Macro used for the automatic quantification of CX3CR1+ and CCR2+ cells in the choroid plexus of CX3CR1-GFP x CCR2-RFP mice. [file DataSheet_1.pdf]

Supplementary Table 1

Target mRNA	Forward Sequence (5´-3´)	Reverse Sequence (5´-3´)	Reference
Aldoa	CCTTAGTCCTTTCGCCTACCC	TGCTTTCCTTTCCTAACTCTGTCT	*Designed with NCBI Primer Blast*
Arg 1	CTCCAAGCCAAAGTCCTTAGAG	AGGAGCTGTCATTAGGGACATC	*(Tomita, Freeman et al. 2016)*
Cd86	TTGTGTGTGTTCTGGAAACGGAG	AACTTAGAGGCTGTGTTGCTG GG	*(Kigerl, Gensel et al. 2009)*
Cd38	TTGCAAGGGTTCTTGGAAAC	CGCTGCCTCATCTACACTCA	*(Lee, Song et al. 2012)*
Ccr1	GAACGGTTCTGGAAGTACCT	TGGTTGACACCTATGGTCTG	*(Khan, Hashimi et al. 2016)*
Ccr2	CAAGGTCATCCATGACAACTTTG	GGCTGCTTTTGTAGGCTTCA GTG	*(Lu and Kang 2009)*
Ccr5	CTGGACTCCCTACAACATTG	ACACTGAGAGATAACTCCGG	*(Khan, Hashimi et al. 2016)*
Egr2	CTACCCGGTGGAAGACCTC	AATGTTGATCATGCCATCTCC	*(Zheng, Zha et al. 2012)*
Fpr2	TCTACCATCTCCAGAGTTCTGTGG	TTACATCTACCACAATGTGAA CTA	*(Jablonski, Amici et al. 2015)*
G-csf	CCTGCAGGCTCTATCGGGTA	CACCCCTAGGTTTTCCATCTGC	*Designed with NCBI Primer Blast*
Gm-csf	ACATGCCTGTCACGTTGAATG	AAATTGCCCCGTAGACCCTG	*Designed with NCBI Primer Blast*
Gpi1	CCTTGCTGCCCTATGACCAG	CATCTTGGTGCCTTGGTGGA	*Designed with NCBI Primer Blast*
Gpr18	CGACCAAGAAAAGAACCACAG	AATGAAAGCAAGAAGCCACA	*(Taylor, Christou et al. 2015)*
Hprt	TGAAGAGCTACTGTAATGATCAG TCAAC	AGCAAGCTTGCAACCTTAACCA	*(Jablonski, Amici et al. 2015)*
Il1b	TGCCACCTTTTGACAGTGATG	GATGTGCTGCTGCGAGATTT	*Designed with NCBI Primer Blast*
Il6	CCTACCCCAATTTCCAATGCTC	GGTCTTGGTCCTTAGCCACT	*Designed with NCBI Primer Blast*
Il6r	TGGAACCCCACACAGGTCTC	CGAGGATTCTTGCACTGGGG	*Designed with NCBI Primer Blast*
Mmp2	CCCCCATGAAGCCTTGTTTAC	ACAGTGGACATAGCGGTCTC	*Designed with NCBI Primer Blast*
Mrc1	CCACAGCATTGAGGAGTTTG	ACAGCTCATCATTTGGCTCA	*(Tatano, Shimizu et al. 2014)*
Nos2	CCGAAGCAAACATCACATTCA	GGTCTAAAGGCTCCGGGCT	*(Lu, Zhang et al. 2015)*
Pfkl	AGTGCCAGAGACTACATTCAGC	GGTCATGTGATCGTGTGAGTG	*Designed with NCBI Primer Blast*
Retnla	CCCTGCTGGGATGACTGCTA	TGCAAGTATCTCCACTCTGGA TCT	*(Han, Kim et al. 2017)*
S16	CACTGCAAACGGGGAAATGG	TGAGATGGATCGGATGG	*Designed with NCBI Primer Blast*

Stat1	CTGAATATTTCCCTCCTGGG	TCCCGTACAGATGTCCATGAT	*(Shaul, Bennett et al. 2010)*
Tpi1	CCCACCGCTTACATCGACTT	ATGCCAGGGCTGATTTCCC	*Designed with NCBI Primer Blast*
Ym1	GGGCATACCTTTATCCTGAG	CCACTGAAGTCATCCATGTC	*(Shaul, Bennett et al. 2010)*

Supplementary Table 1. The forward and reverse primers used for the RTqPCR experiments used in present study.

Supplementary Table 2

Target gene	Mean Cycle Threshold (Ct) Value (± Standard Deviation)		
	$M^{unpolarized}$	$M^{LPS+IFN-\gamma}$	$M^{IL-4+IL-13}$
Hprt	23.35 (±0.77)	23.23 (±0.97)	23.18 (±0.93)
Nos2	31.42 (±3.51)	19.10 (±2.17)	30.55 (±1.74)
Fpr2	26.99 (±0.87)	19.46 (±0.39)	30.31 (±0.49)
Gpr18	27.93 (±1.95)	24.59 (±0.83)	27.49 (±0.66)
Cd86	27.66 (±3.04)	22.51 (±2.84)	28.30 (±2.57)
Cd38	30.22 (±0.61)	22.26 (±0.80)	30.16 (±0.45)
Stat1	23.46 (±0.70)	19.12 (±0.52)	23.07 (±0.75)
Arg 1	31.10 (±0.88)	26.45 (±1.31)	20.34 (±0.89)
Mrc1	22.53 (±0.42)	28.93 (±0.78)	21.04 (±0.03)
Ym1	27.65 (±0.13)	32.24 (±0.68)	18.85 (±1.33)
Retnla	30.67 (±1.49)	30.78 (±0.56)	19.30 (±2.45)
Egr2	23.54 (±1.48)	27.23 (±0.21)	21.83 (±0.92)
Ccr1	24.07 (±0.38)	23.05 (±0.46)	23.40 (±0.54)
Ccr2	23.53 (±1.32)	28.07 (±0.99)	24.47 (±0.84)
Ccr5	20.86 (±0.25)	20.74 (±0.05)	20.78 (±0.28)

Supplementary Table 2. Relative mRNA expression of different pro- and anti-inflammatory markers and chemokine receptor genes expressed by $M^{unpolarized}$, $M^{LPS+IFN}$ and $M^{IL-4+IL-13}$ cells following 48h stimulation. Displayed are the mean and standard deviation of cycle threshold (Ct) values obtained from technical triplicates following RTqPCR reaction from six to seven experiments (for *Hprt, Nos2* and *Arg1*) and from three experiments (for *Fpr2, Gpr18, Cd86, Cd38, Stat1, Mrc1, Ym1, Retnla, Egr2, Ccr1, Ccr2, Ccr5*).

Supplementary Table 3

Target gene	Mean Cycle Threshold (Ct) Value (±Standard Deviation)						
	$M^{unpolarized}$	$M^{Unstimulated}$ Endothelium	$M^{IL\text{-}1\beta}$ Endothelium	$M^{TNF\text{-}\alpha}$ +IFN-γ Endothelium	$M^{Unstimulated}$ Epithelium	$M^{TNF\text{-}\alpha}$ Epithelium	$M^{IFN\text{-}\gamma}$ Epithelium
Hprt	23.29 (±0.35)	23.60 (±0.33)	23.55 (±0.27)	23.70 (±0.46)	23.48 (±0.30)	23.65 (±0.24)	23.91 (±0.75)
Nos2	25.80 (±1.70)	25.37 (±0.77)	24.87 (±0.48)	22.32 (±0.41)	25.97 (±0.53)	25.55 (±1.38)	22.83 (±1.62)
Arg1	30.55 (±0.46)	29.13 (±0.24)	25.67 (±0.43)	28.45 (±0.94)	27.52 (±0.26)	27.75 (±0.17)	29.39 (±1.64)
Tpi1	18.98 (±1.05)	18.83 (±0.69)	18.45 (±0.79)	18.75 (±0.64)	18.58 (±0.45)	18.91 (±0.86)	19.07 (±1.13)
Gpi1	19.72 (±0.96)	19.45 (±0.43)	19.09 (±0.50)	19.52 (±0.44)	19.18 (±0.39)	19.38 (±0.50)	19.82 (±1.45)
Pfkl	25.58 (±0.86)	25.24 (±0.44)	24.89 (±0.56)	25.17 (±0.55)	25.09 (±0.40)	25.30 (±0.51)	25.70 (±1.66)
Aldoa	25.80 (±0.91)	26.06 (±0.35)	25.62 (±0.46)	25.95 (±0.38)	25.49 (±0.24)	25.64 (±0.75)	26.64 (±1.70)
Il6	31.58 (±0.30)	31.55 (±0.17)	31.01 (±0.21)	30.95 (±0.28)	32.01 (±0.57)	31.63 (±0.04)	31.91 (±0.20)
Il6r	23.72 (±0.94)	23.58 (±0.67)	23.10 (±0.70)	23.93 (±0.83)	23.68 (±0.96)	23.76 (±0.75)	24.04 (±1.55)
Mmp2	28.48 (±0.77)	27.95 (±0.10)	27.60 (±0.13)	27.81 (±0.32)	27.61 (±0.24)	27.69 (±0.21)	28.35 (±1.16)
Ilβ	29.15 (±0.78)	26.84 (±0.64)	24.25 (±1.24)	26.67 (±1.74)	27.62 (±0.50)	27.93 (±0.63)	28.42 (±0.67)

Supplementary Table 3. Relative mRNA expression of inflammatory and glycolysis genes expressed by $M^{unpolarized}$ and MoCs incubated with unstimulated and 16h-cytokine stimulated endothelial and epithelial cells following 7h stimulation. Displayed are the mean and standard deviation of cycle threshold (Ct) values obtained from technical triplicates following RTqPCR reaction from three independent experiments.

Supplementary Table 4

Target gene	Mean Cycle Threshold (Ct) Value (±Standard Deviation)		
	Endothelium		
	Unstimulated	IL-1β	TNF-α +IFN-γ
Hprt	23.22 (±1.41)	23.35 (±1.33)	22.76 (±1.36)
Gm-csf	29.17 (±1.24)	28.82 (±0.98)	28.94 (±1.14)
G-csf	31.47 (±0.15)	28.19 (±2.46)	31.21 (±0.75)

Supplementary Table 4. Relative mRNA expression of genes expressed by unstimulated and cytokine stimulated endothelium following 16h stimulation. Displayed are the mean and standard deviation of cycle threshold (Ct) values obtained from technical triplicates following RTqPCR reaction from three independent experiments.

Supplementary Table 5

Target gene	Mean Cycle Threshold (Ct) Value (±Standard Deviation)			
	$M^{Unstimulated-Isotype}$	$M^{IL-1\beta}$ - Isotype	$M^{Unstimulated}$ G-CSF+GM-CSF	$M^{IL-1\beta}$ pMBMECs- G-CSF+GM-CSF
S16	21.71 (±0.36)	21.48 (±0.44)	21.76 (±0.46)	21.00 (±0.23)
Arg1	28.93 (±1.66)	25.97 (±2.13)	29.47 (±1.09)	27.12 (±2.60)
Tpi1	20.18 (±1.04)	19.76 (±0.95)	20.14 (±1.02)	19.58 (±1.03)
Gpi1	21.37 (±0.97)	20.85 (±0.97)	21.33 (±0.74)	21.71 (±1.09)

Supplementary Table 5. Relative mRNA expression of inflammatory and glycolytic genes expressed by MoCs following incubation with unstimulated or 16h cytokine activated endothelial cells. Displayed are the mean and standard deviation of cycle threshold (Ct) values obtained from technical triplicates following RTqPCR reaction from four independent experiments.

Supplementary Table 6

Target gene	Mean Cycle Threshold (Ct) Value (±Standard Deviation)						
		$M^{Unpolarized}$ + G-CSF			$M^{Unpolarized}$ + GM-CSF		
	$M^{Unpolarized}$	10ng	50ng	100ng	10ng	50ng	100ng
Hprt	23.85 (±0.42)	24.06 (±0.30)	23.84 (±0.09)	24.15 (±0.19)	24.05 (±0.17)	23.98 (±0.27)	24.03 (±0.21)
Arg1	29.88 (±0.36)	29.76 (±0.52)	30.13 (±0.40)	29.89 (±0.46)	27.68 (±0.80)	27.26 (±0.80)	27.52 (±0.66)

Supplementary Table 6. Relative mRNA expression of *Arg1* genes expressed following 7h incubation of $M^{unpolarized}$ cells with different concentrations (10ng, 50ng, 100ng) of recombinant mouse G-CSF, GM-CSF proteins. Displayed are the mean and standard deviation of cycle threshold (Ct) values obtained from technical triplicates following RTqPCR reaction from three independent experiments.

Fiji Macro used for the automatic quantification of macrophages attached on the luminal side of BBB endothelial cells or on the upper side of ChP epithelial cells filters *in vitro*

```
directory = getDirectory("Choose a Directory");
fileList = getFileList(directory);
out_dir = directory + "/Analysis/"; //
File.makeDirectory(out_dir);
mask_dir = out_dir + "/Masks/"; //
File.makeDirectory(mask_dir);
results_dir = out_dir + "/Results/"; //
File.makeDirectory(results_dir);
setBatchMode(true);

for (i = 0; i < fileList.length; i++) {
	path = directory + fileList[i];
	open(path);
	Title = getTitle();
	run("Gaussian Blur...", "sigma=1.0");
	run("Brightness/Contrast...");
	setMinAndMax(0, 120);
	setAutoThreshold("Otsu dark");
	run("Threshold...");
	setOption("BlackBackground", false);
	run("Convert to Mask");
	run("Watershed");
	run("Set Measurements...", "area perimeter integrated redirect=None
	decimal=4");
	run("Analyze Particles...", "size=4-Infinity circularity=0.00-1.00
	show=Outlines display summarize");
	selectWindow("Summary");
	saveAs("Results", ""+results_dir+"Summary of " +Title+".csv");
	selectWindow("Drawing of "+Title+"");
	saveAs("tiff", ""+mask_dir+"Mask of " +Title+".tiff");
```

Fiji Macro used for the automatic quantification of CX3CR1^{+} and CCR2^{+} cells in the choroid plexus of ***CX3CR1-GFP x CCR2-RFP*** **mice**

```
directory = getDirectory("Choose a Directory");
fileList = getFileList(directory);
out_dir = directory + "/Analysis/"; //
File.makeDirectory(out_dir);

mask_dir = out_dir + "/Masks/"; //
File.makeDirectory(mask_dir);
results_dir = out_dir + "/Results/"; //
File.makeDirectory(results_dir);
ROIs_dir = out_dir + "/ROIs/"; //
File.makeDirectory(ROIs_dir);

for (i = 0; i < fileList.length; i++) {
			path = directory + fileList(i];
			open(path);
			Title = getTitle();
run("Split Channels");

print(Title);
print("C1-"+Title+"");

selectWindow("C1-"+Title+"");
			waitForUser("Create selection","Make your ROI selection and click OK
			to continue: ");
			roiManager("Add");
			roiManager("Save", ""+ROIs_dir+"ROI of" +Title+".roi");
			run("Clear Outside");
			run("Gaussian Blur...", "sigma=3");
			setAutoThreshold("Otsu dark");
			setOption("BlackBackground", false);
			setThreshold(35, 255);
			title = getTitle();
			run("Convert to Mask");
			run("Erode");
			run("Set Measurements...", "area perimeter shape integrated display
			redirect=None decimal=4");
			run("Analyze Particles...", "size=10-Infinity circularity=0.00-1.00
			show=Outlines display summarize");
			saveAs("Results", ""+results_dir+"Summary of " +title+".csv");
			selectWindow("Drawing of "+title+"");
			saveAs("tiff", ""+mask_dir+"Mask of " +title+".tiff");
			run("Clear Results");

selectWindow("C2-"+Title+"");
			run("Restore Selection");
```

```
                run("Clear Outside");
                run("Gaussian Blur...", "sigma=3");
                setAutoThreshold("Otsu dark");
                setOption("BlackBackground", false);
                setThreshold(35, 255);
                title = getTitle();
                run("Convert to Mask");
                run("Erode");
                run("Set Measurements...", "area perimeter shape integrated display
                redirect=None decimal=4");
                run("Analyze Particles...", "size=10-Infinity circularity=0.00-1.00
                show=Outlines display summarize");
                saveAs("Results", ""+results_dir+"Summary of " +title+".csv");
                selectWindow("Drawing of "+title+"");
                saveAs("tiff", ""+mask_dir+"Mask of " +title+".tiff");
                run("Clear Results");

                imageCalculator("AND create", "C1-"+Title+"", "C2-"+Title+"");
                run("Set Measurements...", "area perimeter shape integrated display
                redirect=None decimal=4");
                run("Analyze Particles...", "size=10-Infinity circularity=0.20-1.00
                show=Outlines display summarize");
                selectWindow("Drawing of Result of C1-"+Title+"");
                saveAs("tiff", ""+mask_dir+"Mask of double positive_ " +Title+".tiff");
                saveAs("Results", ""+results_dir+"Summary of double positive_"
                +Title+".csv");
                selectWindow("Summary");
                saveAs("Results", ""+results_dir+"Results_" +Title+".csv");
}
roiManager("Save", ""+ROIs_dir+"RoiSet.zip");
cleanUp();
function cleanUp() {
   requires("1.30e");
   if (isOpen("Results")) {
       selectWindow("Results");
       run("Close" );
   }
     if (isOpen("Threshold")) {
       selectWindow("Threshold");
       run("Close" );
   }
   if (isOpen("Summary")) {
       selectWindow("Summary");
       run("Close" );
   }
   if (isOpen("Log")) {
       selectWindow("Log");
       run("Close" );
```

```
    }
    while (nImages()>0) {
          selectImage(nImages());
          run("Close");
    }
}
```
